# Tumor microenvironment–targeted PROTAC nanoparticle self-assembly broadly predicted by structural descriptors

**DOI:** 10.1126/sciadv.adu2292

**Published:** 2025-12-05

**Authors:** Kristen C. Vogt, Magdalini Panagiotakopoulou, Mandana T. Manzari Honu, Ana Marie Perea, Xiaoping Hu, Xufen Yu, Raashed Raziuddin, Quincey LaPlant, Stephen Ruiz, G. Praveen Raju, Jian Jin, David A. Scheinberg, Daniel A. Heller

**Affiliations:** ^1^ Memorial Sloan Kettering Cancer Center, New York, NY 10065, USA.; ^2^ Weill Cornell Medicine, Cornell University, New York, NY 10065, USA.; ^3^ Tri-Institutional PhD Program in Chemical Biology, Memorial Sloan Kettering Cancer Center, New York, NY 10065, USA.; ^4^ Mount Sinai Center for Therapeutics Discovery, Departments of Pharmacological Sciences, Oncological Sciences, Tisch Cancer Institute, Icahn School of Medicine at Mount Sinai, New York, NY 10029, USA.; ^5^ Department of Radiation Oncology, Memorial Sloan Kettering Cancer Center, New York, NY 10065, USA.; ^6^ Departments of Neurosciences and Pediatrics, Moores Cancer Center, University of California, San Diego, La Jolla, CA 92093, USA.

## Abstract

Proteolysis-targeting chimeras (PROTACs) are catalytic protein degraders with promising preclinical activity. The clinical translation of PROTACs has been limited by poor pharmacologic properties and toxicities, in part due to their “non-druglike” characteristics, including large molecular weights. We found that the vast majority of PROTACs can self-assemble into nanoparticles, yielding nanoparticle PROTACs (nanoPROTACs) with ultrahigh drug loadings. While PROTAC molecular features can be deleterious to their pharmacokinetic properties, we found that they can drive nanoencapsulation more efficiently than FDA-approved small-molecule drugs. Using structure-based prediction algorithms, we identified spatial autocorrelation molecular descriptors that defined nanoPROTAC formation with 96% sensitivity at 100% specificity. NanoPROTACs, targeted to the tumor microenvironment via P-selectin, led to significantly enhanced tumor drug uptake, target degradation, tumor growth inhibition, and overall survival in solid tumor xenografts. These findings offer a broad strategy to improve the pharmacologic properties and therapeutic index of PROTACs and potentially other non-druglike experimental therapeutics.

## Teaser

Nanoparticles targeted to solid tumor vasculature improve the pharmacologic properties of PROTACs.

## INTRODUCTION

Approximately 85% of the human proteome remains “undruggable” by classical chemical inhibitor-based methods due to a lack of accessible protein binding sites. The proteolysis targeting chimera (PROTAC) approach expands the list of potentially druggable targets by inducing protein degradation via the ubiquitin-proteasome system (*1*). PROTACs are chimeric molecules with two binding moieties: a warhead that binds to the protein of interest (POI) and a ligand that recruits an E3 ubiquitin ligase, a key enzyme in the protein degradation pathway of the cell. PROTACs structurally tether these two moieties together to form a ternary complex (POI-PROTAC-E3 ligase), marking the protein for proteasomal degradation via ubiquitination. Protein degraders can bind any accessible area of a target protein, allowing for previously undruggable proteins to be pharmacologically targeted.

Now, at least 15 PROTACs are under early-stage clinical evaluation in patients (*2*). The PROTACs ARV-110 (NCT03888612) and ARV-471 (NCT04072952), degraders of androgen receptor and estrogen receptor, respectively, have demonstrated safety in patients with prostate and breast cancers. However, recent reports of ARV-471 demonstrated suboptimal efficacy in a phase 2 expansion cohort with only two patients having confirmed partial responses out of 44 subjects with measurable disease (*3*). Similarly, ARV-110 trials confirmed only two of seven patients with partial responses, despite reducing prostate-specific antigen by more than half in 46% of patients (*4*). Together, these trials show the limited success of PROTACs in the clinic to date and highlight the unmet need to improve PROTAC delivery to potentiate their therapeutic efficacy in solid tumors.

The limited success of protein degraders in the clinic may be due in part to the poor pharmacologic properties of PROTACs that can result from their chimeric molecular structure, which are far from the traditional rules for “drug-like” properties defined by Lipinski’s rule of 5 (*5*–*8*). While certain successful therapeutics like taxanes achieve clinical success despite violating Lipinski’s rules through specialized formulation strategies, PROTACs introduce distinct medicinal chemistry hurdles. Notably, these “beyond rule-of-five” drugs are generally much larger (800 to 1300 Da) than most conventional drug-like small molecules (<500 Da). PROTACs also tend to be hydrophobic (log*P*) and have larger numbers of hydrogen bond donors and acceptors (*9*). Thus, many PROTACs exhibit poor bioavailability, cell penetration, and biodistribution in tumors (*10*). Furthermore, PROTACs are not completely selective for tumor cells and can cause on-target toxicities via uptake by healthy tissue (*11*). Although this may be avoided by using cell type– and tumor-selective E3 ligases, there are limited known selective E3 ligases, and the chemical screening and synthesis of such PROTACs are expensive and laborious (*11*).

One promising PROTAC, for instance, is dBET6, one of a family of highly potent bromodomain and extra-terminal domain (BET) degraders that selectively degrade BRD2, BRD3, and BRD4 in the nanomolar range (*12*). BRD4 is a transcriptional coactivator involved in regulation of oncogene expression. Previous work demonstrated significant antitumor effects of dBET6 in preclinical models of leukemia and triple-negative breast cancer by twice daily injections of the drug (*12*, *13*). However, efforts to improve this dosing regimen to daily injections were ineffective and resulted in dose-limiting toxicities (*13*). These complications may be partly attributed to the rapid blood clearance of dBET6 [terminal *t*_1/2_ (half-time) = 0.870 hours] or to potential on-target, off-tumor degradation of the target resulting from systemic administration of the drug (*12*). Nanoscale drug delivery approaches can potentially overcome such pharmacologic challenges by modulating drug pharmacokinetics, stability, absorption, and exposure to tumors and healthy tissues (*14*, *15*). Nanomedicines promise to expand the therapeutic window of the unmodified, or “free,” drug by enhancing tumor uptake through both passive and active targeting while sparing healthy tissue and mitigating toxicities (*16*, *17*). Despite the promise of targeted nanomedicines, clinical translation of these therapeutics has been hindered by the complexity of carrier design and synthesis and low drug loading (*18*, *19*).

Computational approaches to nanocarrier design can facilitate synthesis of drug delivery nanoparticles (NPs) with diverse payloads (*20*–*22*). We found that the encapsulation of hydrophobic drugs into dye-stabilized NPs with ultrahigh loadings (up to 90% drug by mass) could be predicted to form via quantitative structure NP assembly prediction (QSNAP) calculations, resulting in the finding that using two descriptors—hydrophobicity (log*P*) and the number of high intrinsic state substructures (NHISS)—reliably predicted small-molecule drug assembly into NPs (*23*). It is not known, however, whether QSNAP can predict functionalized particle self-assembly for beyond rule-of-five drugs with high degrees of molecular complexity, such as PROTACs. Separately, we found that drugs can be actively targeted to solid tumor vasculature by incorporating fucoidan (Fi), a polysaccharide with nanomolar affinity to P-selectin, a cell adhesion molecule expressed on activated vasculature with minimal expression in normal tissues. These targeted NPs improved the therapeutic index of encapsulated drugs and led to substantial tumor regression, prolonged overall survival, and reduced side effects (*17*, *23*–*26*).

Here, we developed a broadly applicable nanoformulation approach for the targeted delivery of nanoparticle PROTACs (nanoPROTACs) to solid tumors. We found, using the combined log*P* and NHISS (log*P*/NHISS) descriptor, that the combined molecular properties of PROTAC components (E3 ligand linked to a chemical warhead) are largely predicted to drive nanoencapsulation due to high numbers of electronegative substructures and increased hydrophobicity. Specifically, 93% of PROTACs, 24% of chemical warheads, and 12% of E3 ligase recruiters in the PROTAC-DB database were predicted to form NPs, highlighting the unique molecular features of PROTACs to support nanoencapsulation. Experimentally, the log*P*/NHISS descriptor predicted nanoPROTAC formation with 79% accuracy, and incorporating the P-selectin–targeted polysaccharide fucoidan largely held to the log*P*/NHISS-based self-assembly prediction. To investigate an improved algorithm for nanoPROTAC assembly, we calculated a library of more than 1800 two-dimensional (2D) and 3D molecular features through the open-source cheminformatics package Mordred (*27*). We found that 2D autocorrelation descriptors weighted by ionization potential remarkably predicted nanoPROTAC formation at up to 96% sensitivity at 100% specificity, including upon substantial PROTAC linker modification. We further investigated the targeting and antitumor efficacy of two P-selectin–targeted nanoPROTACs encapsulating degraders of BRD4 or MEK1/2. In both settings, we found enhanced tumor localization over normal tissue, efficient target degradation, and significantly improved antitumor efficacy in murine solid tumor models. These collective findings establish nanoPROTACs as a broadly applicable platform to modulate protein degrader pharmacologic properties and potentially improve clinical translation of PROTACs and other non-druglike experimental therapeutics for therapy in solid tumors.

## RESULTS

### Prediction of PROTAC nanoencapsulation with indocyanine dyes in silico

**Fig. 1.**
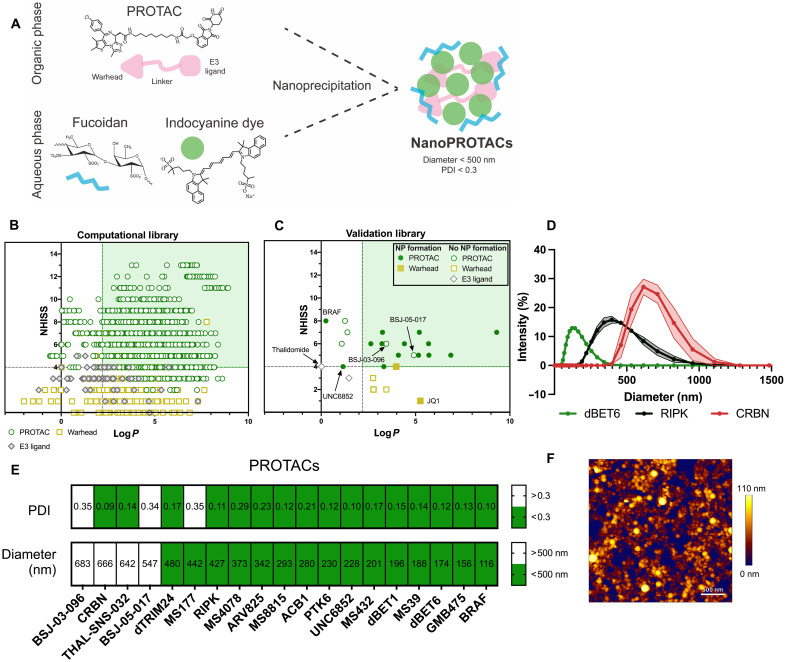
PROTAC molecular properties make them ideal candidates for self-assembly into colloidally stable NPs. (**A**) Nanoformulation scheme of PROTACs by self-assembly with indocyanine dye and fucoidan. (**B**) Propensity for nanoformulation of PROTACs and their structural subunits (warheads and E3 ligase recruiters) obtained from PROTAC-DB, as assessed by hydrophobicity ranking (log*P*) and electronegativity score (NHISS). (**C**) Experimental validation of several PROTACs (*n* = 19), warheads (*n* = 5), and E3 ligase recruiters (*n* = 2), as assessed by log*P* and NHISS. (**D**) Representative DLS profile of PROTACs with varying diameters (*n* = 3). (**E**) Heatmap of polydispersity index (PDI) and diameter (intensity mean) of all nanoPROTACs characterized (green = forms NPs, white = does not form). (**F**) Atomic force micrograph of Fi-dBET6 NPs. Data are individual datapoints [(B) and (C)] or means of technical replicates ± SEM (D).

We first investigated the QSNAP model in the context of PROTAC chemical space to determine the degree to which PROTACs assemble into P-selectin–targeted NPs in the presence of indocyanine dyes or their propensity for nanoformulation (Fig. 1A). We assessed molecular descriptors of PROTACs (*n* = 3621), E3 ligands (*n* = 74), and chemical warheads (*n* = 973) from the database PROTAC-DB to understand differential characteristics of PROTACs and PROTAC building blocks. Specifically, hydrophobicity (log*P*) and the NHISS (a measure of highly electronegative moieties in a molecule) were calculated using the simplified molecular input line entry system (SMILES) strings of each molecule using the online chemical database OCHEM (*28*).

We chose a threshold for structures predicted to form NPs of log*P* > 2.2 and NHISS ≥4, as previously determined for drug-encapsulating NPs stabilized with the indocyanine dye IR783 (*23*). While our nanoPROTAC formulation uses indocyanine green (ICG; or IR-125) in place of IR783, both dyes belong to the sulfated indocyanine dye family and share high structural homology, making this threshold a suitable starting point to inform PROTAC NP formation. Using these criteria, only 24% of PROTAC chemical warheads and 12% of E3 ligands were predicted to form NPs due to either insufficient NHISS or low hydrophobicity (Fig. 1B). Conversely, the log*P*/NHISS ranking was significantly higher for PROTACs, resulting in 93% of PROTACs predicted to form NPs. Connecting two chemical moieties (E3 ligand and chemical warhead) that are individually predicted to not form NPs via a chemical linker can result in a chimeric molecule with combined NHISS and log*P* values that meet the threshold for nanoformulation. In addition, we estimated the ability of small-molecule drugs to form NPs using a library of 11,750 small-molecule drugs from the DrugBank database and found that only 8% of small molecules had sufficient log*P* and NHISS to form NPs. These calculations suggest that the intrinsic molecular properties of PROTACs make them uniquely amenable to a dye-stabilized nanoformulation strategy, despite falling beyond the traditional rule-of-five criteria.

### Synthesis and characterization of nanoPROTACs

We next assessed the predictive capabilities of the log*P*/NHISS method for the self-assembly of a library of nanoPROTACs. We assembled a validation library including PROTACs with diverse structures, as well as unlinked PROTAC components, including E3 ligase ligands (both cereblon and VHL recruiters) and chemical warheads, to assess our computational predictions for NP assembly (Fig. 1C). We found that PROTACs in both libraries had significantly higher NHISS values, as compared to E3 ligands and chemical warheads. Although the observed differences in hydrophobicity did not reach statistical significance, the consistent trend of PROTACs exhibiting greater hydrophobicity across both compound libraries suggests that our selected commercial drug set provides a representative profile of PROTAC-like physicochemical properties (fig. S1).

NanoPROTACs were synthesized using an optimized nanoprecipitation protocol in which PROTACs dissolved in dimethyl sulfoxide (DMSO) were introduced via dropwise addition into an aqueous buffered solution containing the near-infrared (NIR) dye ICG, to stabilize the hydrophobic drug, and the polysaccharide fucoidan, to enable targeting to P-selectin within the tumor microenvironment (Fig. 1A). ICG was selected for NP formulation based on its NIR fluorescent properties and established safety profile as a Food and Drug Administration (FDA)–approved imaging agent (*29*). Following nanoprecipitation, colloidal suspensions were washed by centrifugation, and NP pellets were resuspended in water for assessment in downstream morphological characterization and functional assays.

To benchmark the success of the assembly of each drug into an NP, we established quantitative size thresholds wherein particles with diameters less than 500 nm and a polydispersity index (PDI) less than 0.3 were considered NPs (“formers”), and particles with diameters greater than 500 nm or a PDI greater than 0.3 did not form NPs (“nonformers”) (*30*). This threshold was chosen on the basis of the results of stability testing whereby nanoPROTACs with diameters greater than 500 nm were not colloidally stable, making them ineffective research tools and potential therapeutics (fig. S2). While all PROTACs formed NPs to some degree, the hydrodynamic diameters varied depending on the drug and ranged from 116 to 683 nm (average intensity mean: 357 nm) and PDIs from 0.1 to 0.35 (average PDI: 0.2), as measured by dynamic light scattering (DLS) (Fig. 1, D and E).

We found that the vast majority of PROTACs predicted to form NPs by the log*P*/NHISS criteria did form (86%), except for the CDK4/6 degraders BSJ-03-096 and BSJ-03-096 (Fig. 1C). Two PROTACs in the library predicted not to form NPs (BRAF and UNC6852) did form, despite exhibiting low hydrophobicity, resulting in an overall accuracy of 79%. Most examined E3 ligands and chemical warheads also conformed to the log*P*/NHISS prediction criteria, except for the dBET6 chemical warhead JQ1. These results are largely in agreement with Shamay *et al.* (*23*), where the propensity of kinase inhibitors to assemble into untargeted NPs via log*P*/NHISS was predicted with high accuracy. Furthermore, incorporation of fucoidan did not substantially alter the log*P*/NHISS prediction criteria. Notably, fucoidan functionalization enhanced the stability of 41% of nanoPROTACs, yielding NPs with reduced hydrodynamic diameter and improved monodispersity compared to control formulations lacking fucoidan (fig. S3). Consequently, we prioritized fucoidan-functionalized nanoPROTACs for subsequent cheminformatic and biological studies based on their superior stability and tumor-targeting abilities.

### Cheminformatic identification of molecular features driving nanoPROTAC self-assembly

**Fig. 2.**
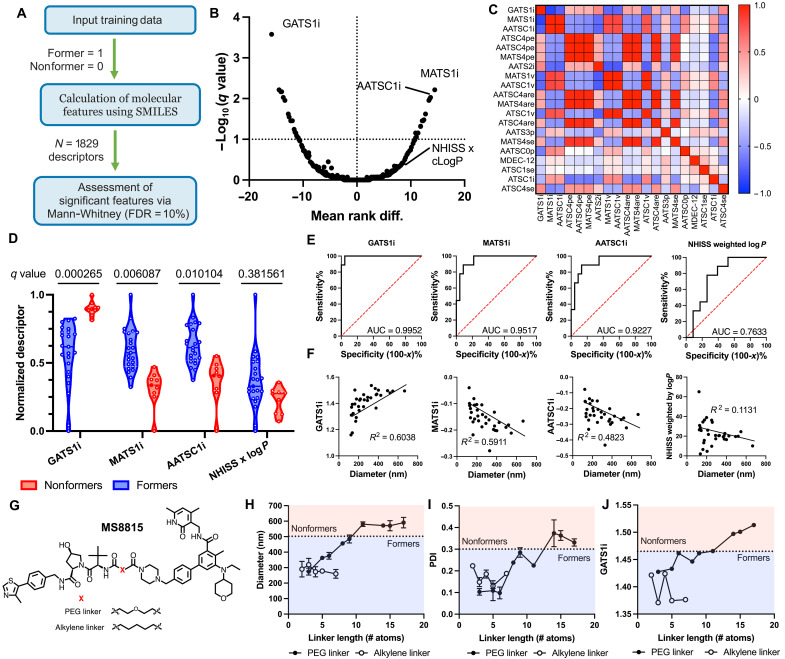
Structural feature analysis reveals molecular drivers of nanoPROTAC formation. (**A**) Workflow for assessment of PROTAC molecular descriptors. (**B**) Volcano plot of all descriptors (*n* = 1829), as calculated by the Mann-Whitney *U* test using a false discovery rate (FDR) of 10%. (**C**) Autocorrelation matrix of top 20 molecular descriptors. (**D**) Top three molecular features describing nanoPROTAC formation, as compared to the log*P*/NHISS model (*n* = 23 formers and 9 nonformers). The library includes the 19 PROTACs in Fig. 1, plus additional 13 PROTAC linker variants (*n* = 32 total). (**E**) Receiver operating characteristic curves and (**F**) NP size correlation for GATS1i, MATS1i, AATSC1i, and NHISS weighted by log*P* (NHISS × log*P*). (**G**) Structure of MS8815 PROTAC linker variants tested. Quantitative structure-NP assembly relationship (SNAR) of MS8815, as reported by NP diameter (**H**), PDI (**I**), and GATS1i (**J**) (*n* = 3). Data are individual datapoints [(B), (D), (F), (H), and (I)] or means of technical replicates ± SEM (H). “*q*” represents the *P* value when adjusted for multiple hypothesis testing. AUC, area under the curve.

To enhance the predictive capability of our algorithm, we developed a cheminformatic framework that also accounts for diverse PROTAC linker strategies. This methodology leverages computed molecular descriptors that quantitatively encode structural and physiochemical properties, effectively transforming chemical structures into numerical data. We calculated more than 1800 Mordred molecular descriptors of PROTACs and assessed for differences between formers and nonformers (Fig. 2A). We found 28 significant descriptors following correction for multiple comparisons and using a false discovery rate threshold of 10% (Fig. 2B). The top 20 descriptors were assessed for high similarity to one another (autocorrelation > 0.8) to consolidate alike features (Fig. 2C). Notably, the top three molecular features (GATS1i, MATS1i, and AATSC1i) all belong to the spatial autocorrelation subclass of molecular descriptors weighted by ionization potential. Specifically, these features describe the level of correlation between two objects in chemical space, also known as molecular connectivity indices (*31*, *32*). For example, GATS1i measures the spatial autocorrelation of ionization potential between adjacent atoms in a molecule. Low GATS1i values indicate a more uniform distribution of ionization potentials across the molecular structure, reflecting a spatially coherent electronic surface. Such uniformity is a hallmark of conjugated, planar systems, which are known to promote intermolecular π-π stacking interactions and nanoaggregation (*30*, *33*, *34*). Notably, the top three descriptors identified from our cheminformatic screen resulted in more significant score differences between formers and nonformers, as compared to log*P*/NHISS (Fig. 2D).

Investigating the ionization-weighted autocorrelation features, we found that MATS1i is inversely related to GATS1i (Pearson’s *R* = −0.87) and highly correlated with AATSC1i (Pearson’s *R* = 0.97). Furthermore, GATS1i resulted in the highest sensitivity (96%) at 100% specificity out of all significant molecular descriptors characterized (Fig. 2E). The observed inverse correlation between GATS1i and the other top descriptors is due to their distinct mathematical formulas. GATS1i uses Geary’s *C* coefficient, which emphasizes atomic dissimilarity in ionization potential, while MATS1i uses Moran’s *I* coefficient, which quantifies atomic similarity. A GATS1i value of 1.465 was found to be a critical threshold at which PROTACs with GATS1i > 1.465 formed NPs (23 of 23) and with GATS1i < 1.465 did not form NPs (9 of 10). This threshold demonstrated exceptional predictive power, yielding an overall accuracy of 97% for NP formation potential. The only exception to this model was the degrader dTRIM24, which was predicted not to form NPs (GATS1i = 1.47) but was within (though close to) the cutoff for formers. The top descriptor also showed a moderate correlation with the NP size [coefficient of determination (*R*^2^) = 0.604], highlighting the utility of GATS1i as a powerful predictor of nanoPROTAC formation.

Given the critical role of chemical linkers on PROTAC function, we next sought to understand the effect of linker composition on NP formation by structure-nanoparticle assembly relationship (SNAR). We tested a library of EZH2-targeted PROTACs (MS8815) with varying linker lengths and compositions [polyethylene glycol (PEG) or alkylene] for their ability to form stable NPs (diameter < 500 nm and PDI < 0.3) (Fig. 2G and fig. S5A) (*35*). We observed a positive correlation between increasing PEG linker length and NP size, with a plateau in size above a PEG linker length of 11 atoms (Fig. 2H). Conversely, increasing the length of alkylene linkers did not affect the NP size. Similar trends in NP PDI were observed in both PEG and alkylene linker types, with larger NPs generally having higher PDI values (Fig. 2I). All MS8815 linker variants conformed to the established GATS1i threshold with 100% accuracy (Fig. 2J). We assessed colloidal stability of each linker variant 5 days postsynthesis and observed that PROTACs with higher GATS1i scores began to precipitate out of solution and were no longer colloidally stable (fig. S5B). To quantify colloidal dispersion, we measured nanoPROTAC turbidity, or the ability of a suspension to scatter light, via sample absorbance. NanoPROTAC linker variants with low absorbance correlated with qualitative observations of NPs precipitating out of solution (fig. S5C). Together, these data further support the use of GATS1i as a positive predictor of nanoPROTAC formation in a library of PROTACs with diverse chemical linkers.

We next selected two representative fucoidan-functionalized, P-selectin–targeted nanoPROTAC formulations for comprehensive in vivo evaluation: Fi-dBET6 and Fi-MS432 NPs. DLS measurements revealed average diameters of ~175 and ~200 nm for Fi-dBET6 and Fi-MS432 NPs, respectively, and a narrow particle size distribution (PDI < 0.2) (Fig. 1E). Furthermore, atomic force microscopy characterization confirmed that both formulations maintained uniform spherical morphologies (figs. S5A and S6A). Most NP preparations achieved higher than 50% drug encapsulation efficiency, as determined by high-performance liquid chromatography (HPLC) (figs. S5B and S6B). Batch-to-batch variability in encapsulation efficiency may be partly attributed to the inconstant flow rate during dropwise nanoprecipitation. Drug loading was remarkably high, with NPs consisting of up to ~90% drug by mass (figs. S5C and S6C). We also synthesized and characterized untargeted control NPs incorporating dextran sulfate (Dex), a polysaccharide with reduced affinity for P-selectin (fig. S5E) (*17*, *24*). Surface functionalization of nanoPROTACs was verified by zeta potential analysis, which showed a negative shift following addition of the negatively charged polysaccharides (figs. S5 and S6). Last, we demonstrated particle stability in both storage (4°C in saline) and physiological conditions (37°C in mouse serum) by DLS and HPLC (figs. S5, F and G, and S6F).

### Assessment of nanoPROTAC functionality and mechanism of action

**Fig. 3.**
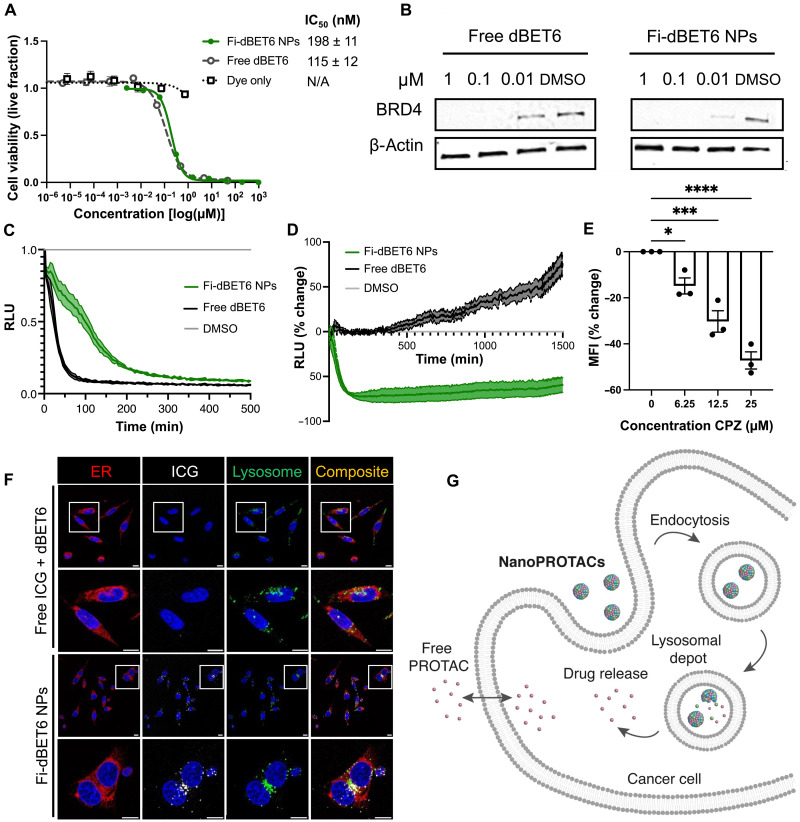
Characterization and mechanistic profiling of Fi-dBET6 NPs by live cell kinetics of target degradation and in vitro NP localization. (**A**) Cell viability curves of NMC cells after treatment with free dBET6 or Fi-dBET6 NPs, as assessed by CellTiter-Glo. Dye-only control was treated at concentrations equivalent to that encapsulated into Fi-dBET6 NPs, as quantified by HPLC. N/A, not available. (**B**) Immunoblotting of BRD4 and β-actin in NMC cells after 24-hour treatment. (**C**) NanoBiT cells were treated with 0.01 μM of either free dBET6 or Fi-dBET6 NPs at *t* = 0. (**D**) NanoBiT cells were treated with free dBET6 or Fi-dBET6 NPs and washed three times with PBS, and the expression of BRD4 was monitored over 24 hours in the presence of Endurazine. Profiles are plotted as mean fractional relative luminescence units (RLU) values by normalizing to DMSO control. (**E**) Mean fluorescence intensity (MFI) of Fi-dBET6 NPs taken up by NMC cells following pretreatment with chlorpromazine (CPZ), a pharmacological inhibitor of endocytosis, as measured by flow cytometry. (**F**) Fi-dBET6 NP uptake and lysosomal colocalization 4 hours postwashout. ER, endoplasmic reticulum. Scale bars, 10 μm. The ROI (white box) is expanded in the second row of each condition. (**G**) Proposed mechanism of nanoPROTAC uptake and release. Data are means of technical replicates ± SEM [(A), (C), and (D)] where *n* = 3 or means of biological replicates ± SEM (E) where n = 3. Statistics were calculated using an ordinary one-way analysis of variance (ANOVA) with Dunnett’s post hoc test (E).

We next investigated the functionality of Fi-dBET6 NPs in vitro using a patient-derived model of NUT midline carcinoma (NMC), a rare and aggressive squamous cell epithelial cancer driven by the BRD4-NUT fusion oncoprotein (*36*, *37*). Specifically, BRD4-NUT maintains tumor growth through a potent chromatin-modifying mechanism (*38*). We found that NMC cells were equally sensitive to free dBET6 and Fi-dBET6 NPs after 24-hour incubation (as measured by CellTiter-Glo viability assay) with ICG dye alone resulting in no measurable cytotoxicity (Fig. 3A). Specific degradation of BRD4 in both free dBET6 and Fi-dBET6 NP–treated cells was observed at nanomolar concentrations by immunoblot (Fig. 3B). Similar cytotoxicity and degradation of BRD4 were observed in Fi-dBET6 NP treatment of MDA-MB-231 triple negative breast cancer cells (fig. S7). These results demonstrate that nanoencapsulation within targeted delivery vehicles preserves the potent degradation capacity and cytotoxic efficacy of dBET6.

We then sought to elucidate the mechanism of action of Fi-dBET6 NPs in cells. The NanoBiT system (Promega) was used to obtain quantitative live cell kinetics of BRD4 degradation. Briefly, NanoBiT-engineered cells express a HiBiT tag on the endogenous BRD4 locus (*39*). LgBiT is stably overexpressed and complements HiBiT to form the luminescent NanoBiT luciferase in the presence of the substrate Endurazine (fig. S8A). Comprehensive degradation profiles were collected by monitoring luminescence over time and fitting the data to a single-component exponential decay model to calculate parameters for the degradation rate, half-life, and plateau (fig. S8, B and C). Fi-dBET6 NP treatment resulted in a unique degradation profile, compared to free dBET6 (Fig. 3C). A delay in BRD4 degradation was observed in Fi-dBET6 NP–treated cells with 3.75-fold increase in time to 50% degradation (*t*_nano_ = 75 min and *t*_free_ = 20 min). While the initial rate of BRD4 degradation was significantly slower in the Fi-dBET6 NP group (*k*_nano_ = 0.009 min^−1^ and *k*_free_ = 0.075 min^−1^), both treatments equilibrated to similar terminal degradation values (Plateau_nano_ = 0.06 and Plateau_free_ = 0.075). These data demonstrate that the initial kinetic differences do not affect the overall ability of dBET6 to degrade BRD4, as previously demonstrated by immunoblot. Rather, the slower degradation profile of Fi-dBET6 NPs may highlight mechanistic differences in drug uptake and bioavailability, potentially signifying a delay in the release of active drug from the NP through the endolysosomal pathway.

To further validate the hypothesis that NP drug availability was slowed by endolysosomal trapping, we assayed for the rebound of luminescent signal via washout studies. NanoBiT cells were treated for 2 hours with either free dBET6 or Fi-dBET6 NPs, washed with phosphate-buffered saline (PBS), and monitored for the BRD4 expression (Fig. 3D). The luminescent BRD4 signal increased after washout of free dBET6, while the signal from Fi-dBET6–treated cells continued to decrease, suggesting extended degradation of BRD4postwashout (Fig. 3D). We measured the concentration of dBET6 at early time points by liquid chromatography–mass spectrometry (LC-MS) and found no significant differences between free dBET6 and Fi-dBET6 NP groups (fig. S8D), suggesting that the different rates of BRD4 degradation were not likely a result of differences in the rate of drug uptake between NP and free drug groups. However, additional studies are warranted to fully elucidate the mechanisms of nanoPROTAC internalization and intracellular trafficking.

We further assessed endolysosomal trafficking by pretreating NMC cells with chlorpromazine (CPZ), a pharmacological inhibitor of clathirin-mediated endocytosis (Fig. 3E). We found that pretreating cells with CPZ led to decreased uptake of Fi-dBET6 NPs, as assessed by reduced mean fluorescence intensity (MFI) of ICG signal via flow cytometry. We further assessed endolysosomal sequestration by imaging NP subcellular localization in cells by confocal microscopy (Fig. 3F). We first treated cells with either Fi-dBET6 NPs or equivalent concentrations of free ICG mixed with free dBET6. We then stained live cells for the lysosome, as well as other subcellular compartments, including the nucleus and endoplasmic reticulum. Fi-dBET6 NPs, but not free ICG dye or free dBET6 controls, colocalized with the lysosome postwashout, suggesting that nanoPROTACs were endocytosed by target cells and trafficked via the endolysosomal pathway, where the drug was slowly released and interacted with the target via diverse decay kinetics, as compared to the free drug (Fig. 3G).

### Targeted nanoPROTACs improve tumor targeting and antitumor efficacy in vivo

**Fig. 4.**
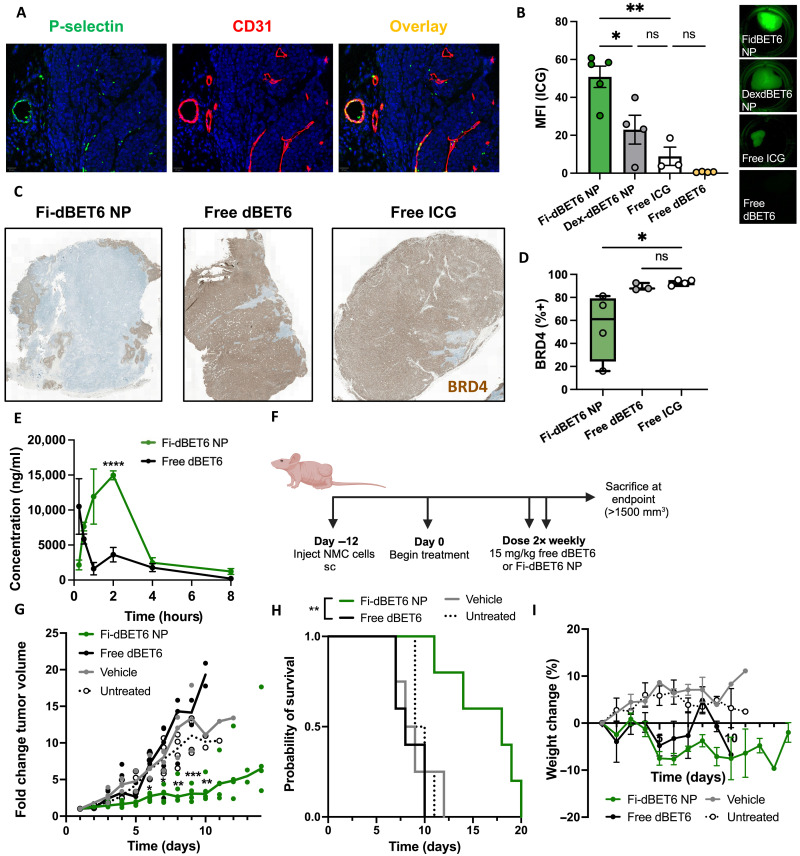
Fi-dBET6 NPs improve antitumor efficacy in NMC xenografts. (**A**) IF staining of P-selectin and CD31 in NMC tumor tissue. (**B**) Quantification of MFI (left) and representative fluorescence emission (right) of NP localization in tumors 24 hours postinjection of Fi-dBET6, Dex-dBET6 (untargeted control), free ICG, or free dBET6 (unlabeled). (**C**) Representative IHC of BRD4 in NMC tissue 48 hours posttreatment. (**D**) Quantification of BRD4 degradation in (C). (**E**) Pharmacokinetics of dBET6, as measured in plasma over time (data are means ± SEM where *n* = 4 biological replicates). (**F**) Nude mice engrafted subcutaneously with NMC cells were treated twice weekly with 15 mg/kg ip treatments of free dBET6, Fi-dBET6 NPs, vehicle, or untreated. (**G**) Tumor growth curves, (**H**) Kaplan-Meier survival curve, and (**I**) mouse weight change in NMC xenografts. Data are shown as individual biological replicates with means ± SEM, and statistics were calculated using one-way ANOVA with Dunnett’s post hoc test [(B) and (D)], multiple unpaired *t* tests with Holm-Sidak correction (E), unpaired *t* test of Fi-dBET6 versus free dBET6 (G), or Mantel-Cox survival analysis (H). ns, not significant; sc, subcutaneous.

We next established a solid tumor model of patient-derived NMC to assess the biodistribution and antitumor efficacy of Fi-dBET6 NPs. NMC cells were engrafted subcutaneously in nude mice and treated or harvested at 100 mm^3^, as measured by digital caliper. P-selectin expression was verified in murine xenografts through immunofluorescence (IF) staining, demonstrating strong colocalization with CD31-positive vascular endothelial cells (Fig. 4A). P-selectin was also observed in primary human NMC tumors, although the specific cell type was not determined (fig. S9). Tumor uptake was then assessed 24 hours posttreatment with free ICG dye, free dBET6 (unlabeled), Fi-dBET6 NPs, or control Dex-dBET6 NPs. We observed significantly higher uptake of targeted Fi-dBET6 NPs, as compared to free ICG dye, in tumors via ex vivo measurements of encapsulated dye MFI (Fig. 4B and fig. S10B). Conversely, there was no significant accumulation of untargeted Dex-dBET6 NPs. Healthy tissues (heart, lung, spleen, and kidneys) showed minimal uptake compared to tumor localization, except for the liver, which is often observed in NP biodistribution studies due to nonspecific uptake by mononuclear phagocytes and hepatic clearance (fig. S10B). These findings are supported by the low expression levels of P-selectin observed in the endothelia of normal tissue (fig. S11).

To validate the correlation between nanoPROTAC biodistribution via ICG signal and dBET6 concentration, we directly measured drug uptake in tumor tissue by LC-MS (fig. S10D). We found that Fi-dBET6 NPs led to the highest concentration of dBET6 in tumors, as compared to untargeted control NPs and freely formulated dBET6. These data suggest that the nanoPROTACs remain intact in vivo and that P-selectin targeting improves delivery of the encapsulated drug to the tumor tissue. Last, we investigated the functional consequence of increased dBET6 within the tumor by immunohistochemistry (Fig. 4, C and D). Fi-dBET6 NPs efficiently degraded BRD4 in tumors after a single 15 mg/kg intraperitoneal (ip) dose. Free dBET6, however, did not achieve significant degradation of BRD4 compared to free ICG control, possibly due to rapid blood clearance, as reported previously (*12*). To further investigate the impact of nanoformulation on dBET6 pharmacokinetics, we assessed blood concentration of dBET6 in healthy mice (Fig. 4E). We found that Fi-dBET6 NPs achieved prolonged plasma exposure, increasing the half-life of dBET6 from 30 min to 3 hours upon nanoencapsulation. These data suggest that our nanoformulation approach enhances tumor exposure to dBET6 by both active targeting to P-selectin and prolonged systemic circulation.

Antitumor efficacy of Fi-dBET6 NPs was then assessed in NMC tumor–bearing mice. Mice were dosed twice weekly with free dBET6 (15 mg/kg; in 40% Captisol), Fi-dBET6 NP, or vehicle (DMSO in 40% Captisol) and monitored for tumor growth via digital calipers (Fig. 4F). Fi-dBET6 NP–treated mice showed a significant delay in tumor growth and extension in overall survival compared to free dBET6 (Fig. 4, G and H). There was no significant antitumor benefit of free dBET6 versus vehicle, supporting previous reports that demonstrate twice daily dosing of dBET6 is required for antitumor efficacy. We observed less than 10% weight loss in both free drug and nanoformulated groups, indicating minimal overall toxicity (Fig. 4I and fig. S12). Tumor growth inhibition studies were repeated in this model and yielded consistent efficacy results (fig. S12).

To demonstrate the broad utility of the nanoPROTAC platform, we selected MS432, a first-in-class degrader of MEK1 and MEK2 (MEK1/2), for evaluation (*40*). MEK1/2 are key signaling proteins in the RAS-RAF-MEK-ERK pathway, and hyperactivation of ERK signaling is associated with 30% of human cancers (*41*). MS432 exhibits low nanomolar DC_50_ (the concentration at which 50% of protein is degraded) in both HT-29 and SK-MEL-28 cells and high selectivity for MEK1/2, as previously determined by global proteomic profiling (*40*). This degrader was not heretofore assessed in vivo for degradation functionality or antitumor efficacy.

We first validated Fi-MS432 NP functionality in vitro by cytotoxicity assays and quantification of MEK1/2 target protein degradation. Treating HCT116 colorectal cancer cells with either free or Fi-MS432 NPs resulted in similar cytotoxicity [median inhibitory concentration (IC_50_) _nanoMS432_ = 0.783 ± 0.06 μM; IC_50 Free MS432_ = 0.783 ± 0.1 μM], as measured by CellTiter-Glo after 24-hour treatment (fig. S13A). Immunoblotting HCT116-treated cells showed time-dependent degradation of MEK1/2 and pMEK1/2 and no significant difference between free and nanoformulated groups over the 36-hour treatment window (fig. S13B). There was a delay in target degradation in Fi-MS432 NP–treated cells, suggesting extended release of the drug, as previously described for Fi-dBET6 NPs. These data demonstrate that nanoformulation of MS432 does not impair or inhibit cytotoxic or functional ability of the encapsulated drug.

To assess Fi-MS432 NPs in vivo, we first evaluated the tolerability of MS432 in the healthy mice at three dose levels (12.5, 25, and 40 mg/kg) to enable dose selection. We found no systemic toxicities at any dose level for either free MS432 or Fi-MS432, as demonstrated by no observed weight loss over the duration of the study (fig. S14). Furthermore, no changes were observed in complete blood count or clinical chemistry panels, relative to the vehicle control, suggesting a lack of blood, liver, and kidney toxicities (figs. S15 and S16). We selected the second dose level (25 mg/kg) for all biodistribution and tumor growth inhibition studies.

**Fig. 5.**
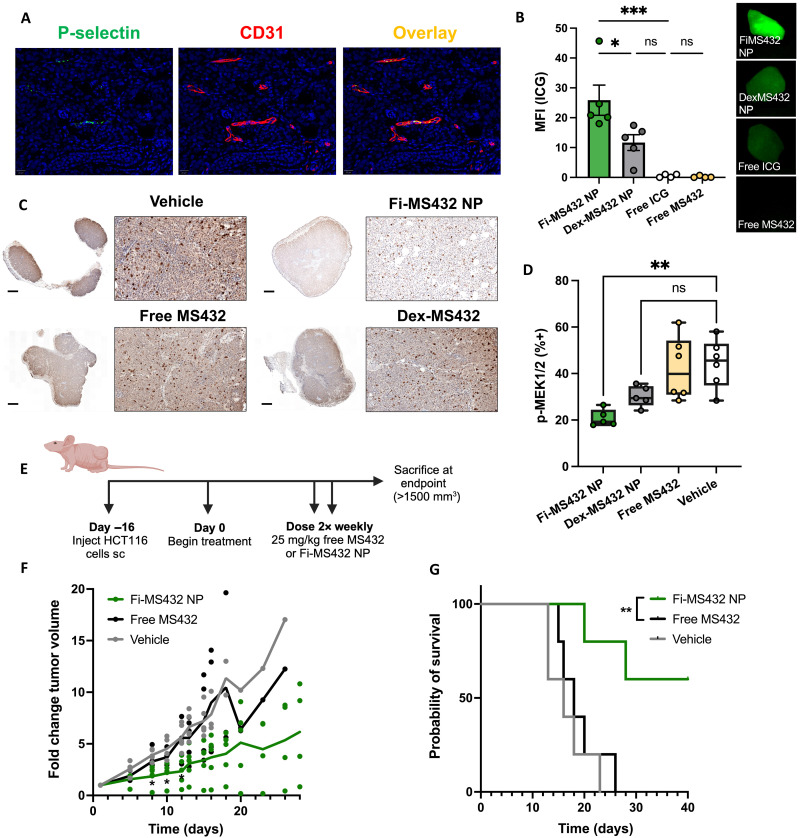
Fi-MS432 NPs improve antitumor efficacy in colorectal cancer xenografts. (**A**) IF staining of P-selectin and CD31 in HCT116 tumor tissue. (**B**) Quantification of MFI (left) and representative fluorescence emission (right) of NP localization in tumors 24 hours postinjection of Fi-MS432, Dex-MS432 (untargeted control), free ICG, or free MS432 (unlabeled). Raw images are reported in fig. S17. (**C**) Representative IHC of p-MEK1/2 in tumor tissue 24 hours posttreatment. (**D**) Quantification of p-MEK1/2 positive cells as shown in (C). (**E**) Nude mice engrafted subcutaneously with NMC cells were treated twice weekly with 15 mg/kg ip treatments of free MS432, Fi-MS432 NP, or vehicle control. (**F**) Tumor growth curves of HCT116 xenografts as assessed by digital calipers. (**G**) Kaplan-Meier survival curve from HCT116 tumor–bearing mice. Data are shown as individual biological replicates with means ± SEM, and statistics were calculated using one-way ANOVA with Dunnett’s post hoc test [(B) and (D)], unpaired *t* test of Fi-MS432 versus free MS432 (F), or Mantel-Cox survival analysis (G).

To evaluate the therapeutic efficacy of Fi-MS432 NPs, we first established HCT116 colorectal adenocarcinoma xenografts in nude mice. IF analysis confirmed P-selectin expression specifically within the tumor vasculature, as demonstrated by colocalization with CD31-positive endothelial cells (Fig. 5A). Biodistribution was then assessed 24 hours posttreatment with free ICG dye, free MS432 (unlabeled), Fi-MS432 NPs, or control Dex-MS432 NPs to assess P-selectin–specific targeting. We observed significantly higher uptake of targeted Fi-MS432 NPs in tumors via ex vivo measurements of encapsulated dye (Fig. 5B and fig. S17). Conversely, there was no significant accumulation of untargeted Dex-MS432 NPs, as compared to the free dye control, highlighting the advantage of active targeting to P-selectin. Normal tissues (heart, lung, spleen, liver, and kidneys) showed minimal retention of the NPs relative to the tumor at later time points (72 hours posttreatment), suggesting a safety profile of Fi-MS432 NPs without substantial concerns (fig. S18). We observed reduced liver uptake of Fi-MS432 NPs compared to Fi-dBET6 NPs (figs. S10 and S18), potentially attributable to: (i) formulation-dependent size and charge differences, (ii) variability in tumor vascularization and P-selectin expression, and (iii) differential dosing (15 and 25 mg/kg, respectively).

We next assessed the pharmacologic consequence of increased intratumoral MS432 concentration on target expression. We found that Fi-MS432 NPs resulted in significantly greater degradation of phosphorylated-MEK1/2 after a single dose, as compared to untargeted control NPs and free MS432 (Fig. 5, C and D). Last, we wanted to determine whether improving target knockdown within the tumor would result in improved antitumor efficacy. HCT116 tumor–bearing mice were treated with free MS432, Fi-MS432 NPs, or vehicle (DMSO) control twice weekly, and tumor growth was monitored via digital calipers (Fig. 5E). We observed a significant therapeutic benefit of Fi-MS432 NP in both delayed tumor growth and improved overall survival, as compared to free MS432 (Fig. 5, F and G).

## DISCUSSION

PROTACs have emerged as an exciting new class of small molecules that can degrade target proteins via the ubiquitin-proteasome pathway. However, their clinical translation has been limited by their pharmacologic properties, including high molecular weight and hydrophobicity, that contribute to low bioavailability, poor cellular penetration, and limited distribution in tumor cells and tissues. In this work, we investigated a potential means to overcome the current pharmacological limitations of PROTACs by assessing their propensity for nanoformulation, evaluating the molecular determinants of NP self-assembly, investigating NP uptake into cells, and assessing tumor microenvironment-targeted delivery of the nanoPROTACs to solid tumors.

To assess whether PROTACs would be amenable to nanoformulation, we investigated the propensity for PROTACs to self-assemble into NPs with high drug loading. We

calculated the combined log*P*/NHISS of PROTACs and their chemical building blocks (E3 ligand recruiters and chemical warheads) using a computational library from the PROTAC-DB database. We first chose hydrophobicity (log*P*) and NHISS as the two main descriptors to classify molecules as predicted to form NPs based on their ability to predict self-assembly of small molecules using indocyanine dye excipients. Notably, on the basis of these two descriptors, the vast majority of PROTACs (93%) were predicted to form NPs, as compared to only 24% of warheads and 12% of E3 ligase recruiters. We also found that PROTACs are substantially more likely to form NPs, as compared to other drug molecules, as demonstrated by an analysis of ~12,000 small-molecule drugs from the DrugBank computational database where only 8% of total compounds were predicted to form NPs. This latter result is in relative agreement with Shamay *et al.*, where less than 6% of FDA-approved drugs were predicted to form NPs.

We validated these findings by synthesizing a library of self-assembled nanoPROTACs stabilized by the indocyanine dye ICG and functionalized with fucoidan to target NPs to tumors positive for P-selectin, a cell adhesion molecule overexpressed on vasculature during inflammation. While both the NP drug core and fucoidan are negatively charged, functionalization can still occur through nonelectrostatic interactions, including hydrophobic interactions, hydrogen bonding, and steric stabilization. For instance, hydrophobic domains of the PROTAC-rich NP core and the relatively amphiphilic regions of fucoidan can facilitate adsorption during nanoprecipitation. ICG was selected for formulations over other indocyanine dyes (such as IR783 and IR800) due to its current FDA approval and clinical use as a NIR imaging agent. We found that the vast majority of PROTACs predicted to form NPs did form, supporting the log*P*/NHISS self-assembly criteria. These findings indicate that the unique molecular features of PROTACs, while often deleterious to their pharmacologic properties as free drugs, can enable formulation into NPs that can potentially obviate the poor pharmacologic properties of their cargo.

While log*P*/NHISS provided rationale for nanoPROTAC assembly, it only accurately categorized 79% of PROTACs as formers or nonformers in our experimental library. To better understand additional drivers of nanoPROTAC formation, we analyzed more than 1800 2D and 3D molecular descriptors. We found substantial positive predictive value from spatial autocorrelation descriptors weighted by ionization potential. Given that each atom in a molecule has an intrinsic ionization potential, molecules with high spatial autocorrelation can be considered spatially homogeneous (such as consecutive carbon chains). The molecular descriptor with highest NP formation correlation, GATS1i, is weighted by Gearson’s *C*, a measure of spatial dissimilarity. Consequently, molecules with low GATS1i exhibit higher spatial similarity. Molecules with more homogeneous spatial electronic properties—as captured by low GATS1i—are better able to engage in π-π stacking (either through electrostatic or charge fluctuation interactions) (*33*, *42*), promoting efficient molecular packing and NP formation. Thus, we propose that GATS1i indirectly reflects molecular features (electronic surface uniformity and planarity) that enhance self-assembly. GATS1i correctly classified nanoPROTAC formation with 96% specificity at 100% sensitivity, as compared to the log*P* and NHISS combined descriptor, which demonstrated only 48% specificity at 100% sensitivity.

We next conducted a SNAR study of PROTAC linker variants to systematically assess the impact of spatial topology and composition on self-assembly. We found that incorporation of a PEG linker introduced a higher degree of spatial heterogeneity (resulting in higher GATS1i), as compared to alkylene linkers of the same length. Together, these studies identified unique molecular features based on spatial similarity that describe nanoPROTAC formation with high accuracy, enabling future formulation studies. The above findings indicate that the unique molecular features of PROTACs, while often deleterious to their pharmacologic properties as free drugs, can enable formulation into NPs that can potentially obviate the poor pharmacologic properties of their cargo.

To evaluate the therapeutic potential of P-selectin–targeted nanoPROTACs, we assessed their cytotoxic function in vitro and in vivo. Live cell assays revealed moderately slower BRD4 degradation kinetics in nanoPROTACs, as compared to the free dBET6 and pretreating cells with pharmacological inhibitors of endocytosis, such as CPZ, blocked uptake of the NPs. These data, in combination with confocal subcellular imaging, highlight the unique mechanism of NP uptake through the endolysosomal pathway and suggest delayed cargo release. Additional systematic characterizations of nanoPROTAC transport mechanisms may help further establish the relationship between drug internalization and therapeutic efficacy.

Last, we found that P-selectin–targeted nanoPROTACs encapsulating degraders of BRD4 and MEK1/2 led to enhanced tumor localization over normal tissue, efficient target degradation, and significantly improved antitumor efficacy in murine solid tumor models. Collectively, these data demonstrate the potent antitumor efficacy of nanoPROTACs in solid tumors, mediated through enhanced intracellular delivery, improved pharmacokinetic profiles, and P-selectin–mediated tumor targeting. Further investigation of nanoPROTAC kinetics in cells and animals could reveal additional mechanisms through which these therapeutics act, such as delayed lysosomal release or sustained intratumoral retention.

Our studies found that an indocyanine dye–based nanoencapsulation strategy presents an avenue to improve the pharmacologic properties of PROTACs, which exhibit several pharmacologic challenges. Preclinically, nanoencapsulation of PROTACs with poor cellular and tissue accessibility may facilitate cell and animal studies and reduce the burden of medicinal chemistry work to conduct preclinical mechanistic studies (*43*). In the clinic, PROTAC nanoformulations may overcome similar pharmacologic tissue barriers to improve drug exposure in diseased tissues and reduce toxicities by targeted delivery strategies, such as demonstrated herein via P-selectin targeting. The possibility to encapsulate nearly any PROTAC with high efficiency and selectively deliver these drugs to solid tumors represents a notable advancement in the field of drug delivery by overcoming the traditional rules that govern drug development.

## MATERIALS AND METHODS

### Reagents

MS432 and MS8815 were synthesized in the laboratory of J.J. (Mount Sinai, Ichan School of Medicine). dBET6 was obtained from Selleck Chemicals. All drugs were dissolved in DMSO. The HiBiT-HaloTag-BRD4 CRISPR cell line in a human embryonic kidney (HEK) 293 background was provided by Promega.

### Computational analysis

A computational library of PROTACs (*n* = 3622), E3 ligands (*n* = 74), and chemical warheads (*n* = 973) was obtained from the PROTAC-DB (*44*). SMILES strings and log*P* values were extracted from PROTAC-DB records. NHISS values were calculated by summing up all the highly electronegative substructures from SMILES strings. NHISS versus log*P* values were then evaluated to determine predicted NP formers with NHISS ≥ 4 and log*P* > 2.2, as previously determined (*23*).

$$\text{NHISS}=n_{\text{fluorine}}+n_{\text{carbonyl}}+n_{\text{sulfinyl}}+2n_{\text{sulfonyl}}+n_{\text{nitroso}}+2n_{\text{nitro}}$$

We then assigned each nanoPROTAC to a binary classification where NP formers (diameter < 500 nm and PDI < 0.3) are assigned to one, and nonformers (diameter > 500 nm or PDI > 0.3) are assigned to zero. SMILES strings were generated for the training data (*n* = 33 PROTACs), and then Mordred was used to calculate 2D and 3D molecular descriptors (*n* = 1829) (*27*). RDKit was used for structure optimization. Each molecular descriptor was then assessed for significant differences between formers and nonformers using a Mann-Whitney test *U* test and Benjamini, Krieger, and Yekutieli correction for multiple comparisons with a false discovery rate of *Q* = 10%.

### Preparation of NPs

An aliquot of 0.05 ml of PROTAC (dBET6, MS432, etc.) dissolved in DMSO (20 mg ml^−1^) was added dropwise to an aqueous solution containing 225 μl of fucoidan (20 mg ml^−1^), 100 μl of ICG (2 mg ml^−1^), and 100 μl of sodium bicarbonate buffer (0.1 M). The resultant suspension was then centrifuged (20 min, 15,000 rpm), and the NP pellet was resuspended in 100 μl of water or 0.9% saline solution (Sigma-Aldrich, #S8776).

### NP characterization

DLS and zeta potential measurements were conducted in water using a Zetasizer Nano ZS (Malvern). Hydrodynamic diameters measured by DLS are reported as intensity mean, unless stated otherwise. Atomic force microscopy was conducted using an Asylum Research MFP-3D-BIO (Molecular Cytology Core Facility, Memorial Sloan Kettering).

PROTAC encapsulation efficiency was quantified by HPLC using C18 analytical columns (150 mm by 2.1 mm; internal diameter, 3.5 μm; Agilent Technologies) with a mobile phase of acetonitrile and deionized water, both with 0.1% trifluoroacetic acid. The gradient ran from 5 to 90% acetonitrile for 5 min and 90 to 95% acetonitrile for 3 min with a flow rate at 1 ml min^−1^. Absorbance values of 256 and 280 nm were used to calculate the area under the curve values for dBET6 and MS432, respectively.

Quantification of fucoidan concentration in NP preparations was determined by Waters via Acquity H-Class System using an Acquity UPLC BEH 450 SEC column (2.5 μm 4.6 × 150 mm P/N 18600685). Briefly, 10 μl of sample was run at a flow rate of 0.3 ml/min using ammonium formate (50 mM) as the mobile phase and water/acetonitrile (50/50) as the wash solvent. Analytes were then detected by evaporative light scattering detector (gas = 45 psi (310.3 kPa), nebulizer = cooling, drift tube temp = 50°C, gain = 500) with a total run time of 12 min. Drug loading and encapsulation efficiency were determined by HPLC.

$$\text{Drug loading}\;\left(\%\right)=\left(\frac{\text{mass drug}}{\text{mass drug}+\text{mass}\;\text{dye}+\text{mass fucoidan}}\right)*100$$

$$\text{Encapsulation efficiency}\;\left(\%\right)=\left(\frac{\text{mass drug in final}\;\text{NP}\;\text{preparation}}{\text{mass drug input}}\right)*100$$

### Stability studies

NanoPROTACs were incubated in media supplemented with 25% mouse serum at 37°C. The amount of released drug was determined by centrifuging NP suspension (10 min, 20,000*g*) at various time points (0, 3, 6, 24, and 48 hours) and extracting drug from the supernatant in 200 μl of ethanol/acetonitrile (1:1). The precipitate was filtered through a 0.2-μm membrane, and drug concentration was quantified using HPLC. Size was quantified at each time point using DLS.

### Cells and cell culture

NMC cells were provided by the laboratory of C. French (Dana-Farber). Other cell lines (HCT116, SKOV3, etc.) were obtained directly from American Type Culture Collection. All cells were maintained in humidified incubators at 37°C in Dulbecco’s modified Eagle’s medium (DMEM) supplemented with 10% heat-inactivated fetal bovine serum (FBS), penicillin (20 U ml^−1^), and streptomycin (20 μg ml^−1^).

### Cell viability

NMC or HCT116 cells (2 × 10^4^) were seeded in a white 96-well tissue culture plate and incubated overnight at 37°C, 5% CO_2_. NPs were added at equivalent drug concentrations in triplicate. Cell viability was assayed by CellTiter-Glo (Promega) 72 hours posttreatment by adding 100 μl of CellTiter-Glo reagent and equilibrating for 30 min. Total luminescence was quantified at 1-s integration time using a Tecan iControl infinite plate reader and normalized to control wells (DMSO treated).

### Live cell kinetics of BRD4 degradation

The HiBiT-HaloTag-BRD4 CRISPR cell line in a HEK293 cell line background stably expressing LgBiT was provided by Promega. HiBiT cells were seeded in white 96-well tissue culture plates and incubated overnight at 37°C, 5% CO_2_ in L-15 CO_2_-independent medium supplemented with 10% FBS. Cells were treated with 20 μM Endurazine for 2.5 hours before addition of a serial dilution of Fi-dBET6 NPs or freely formulated dBET6 in triplicate. Plates were sealed with optical tape and read every 5 min for 24 hours at 1-s integration time using a Tecan iControl infinite plate reader.

### Confocal imaging

NMC cells were seeded on poly-d-lysine–coated chambered cover glass (Thermo Fisher Scientific) and allowed to adhere overnight. Cells were treated with 1 μM Fi-dBET6 NPs or free ICG dye mixed with free dBET6 and incubated at 37°C, 5% CO_2_ for 1.5 hours. Cells were then washed three times with PBS and stained with either LysoTracker green DND-26 (Thermo Fisher Scientific) or ER Tracker Red (Invitrogen) and Hoechst 33342 dye (Thermo Fisher Scientific) in serum-free DMEM. Cells were then imaged 4 hours postwashout using a Leica-SP8 point-scanning confocal microscope with at least three regions of interest per condition.

### Immunoblotting

Cells were lysed using radioimmunoprecipitation assay buffer supplemented with protease inhibitor cocktail for 2 hours at 4°C. The lysates were centrifuged at 13,000 rpm for 5 min at 4°C, and protein concentration was determined using the Bradford assay. Thirty micrograms of protein was loaded into Criterion 4-20% TGX gels (Bio-Rad), resolved via electrophoresis, and then transferred to polyvinylidene difluoride membranes (Bio-Rad). The following primary antibodies were used in this work: BRD4 (E2A7X), β-actin (D6A8), MEK 1/2 (D1A5), pMEK1/2 (41G9), and ERK 1/2 (9102) from Cell Signaling Technology. Blots were blocked for 2 hours in tris-buffered saline blocking buffer and incubated at 4°C overnight with 1:1000 primary in antibody diluent (LI-COR). Blots were washed three times in TBST and incubated with secondary goat anti-mouse immunoglobulin G (IgG) horseradish peroxidase (HRP) conjugate (1:10,000; Abcam) for 1 hour at room temperature. Blots were then incubated with HRP Crescendo substrate for 5 min and imaged using a digital imager (LI-COR).

### Flow cytometry

NMC cells were seeded at a density of 1 × 10^6^ cells/ml in 100 μl of complete DMEM in a 96-well round-bottom plate. Cells were pretreated with the endocytosis inhibitor CPZ at varying concentrations (6.25 to 25 μM) for 1 hour at 37°C and 5% CO_2_. Cells were then treated with 1 μM Fi-dBET6 NPs. After 30 min, cells were washed twice with 200-μl fluorescence-activated cell sorting (FACS) buffer (PBS + 2% FBS + 0.1% sodium azide) and resuspended in 50-μl FACS buffer with 4′,6-diamidino-2-phenylindole (DAPI) and analyzed on a CytoFLEX LX using NIR laser excitation (808 nm).

### Immunohistochemistry

Immunohistochemical staining of human and murine tissue was performed at the Molecular Cytology Core Facility at Memorial Sloan Kettering Cancer Center (MSKCC), as previously described (*45*). Briefly, tissue was harvested and fixed in 4% paraformaldehyde overnight (at 4°C). Tissue was then dehydrated in 70% ethanol for 48 hours, embedded in paraffin, and mounted on microscope slides. Antigen retrieval was performed by standard methods in CC1 buffer (Ventana, no. 950-500), and sections were blocked for 30 min in a background blocking agent (Innovex NB306). Slides were then stained using a Discovery XT processor (Ventana Medical Systems-Roche) using primary antibodies against BRD4 (Bethyl, 50-156-1488), murine CD31 (Abcam, #ab182981), murine P-selectin (LSBio, #LSB3578) or human P-selectin (LSBio, #LSC78725). For immunohistochemistry (IHC), samples were incubated with biotinylated goat anti-rabbit IgG secondary antibody (Vector Labs, no. PK6101) and detected streptavidin-HRP and DAB substrate. For IF, samples were incubated with fluorescent anti-rabbit secondary antibodies (Alexa Fluor 488 and Alexa Fluor 647), counterstained with DAPI, and sealed with a coverslip. Slides were digitally scanned with a digital Panoramic Slide Scanner (3DHistech).

### Mass spectrometry

LC-MS analysis of dBET6 was conducted using an Agilent, Zorbax Eclipse XBD-C18 column; dimensions (2.1 mm by 50 mm, 3.5 μm) on an Agilent triple quadrupole LCMS-6495D. Protocol validation determined drug multiple reaction monitoring transitions: 841.3 → 341, and 841.3 → 383. Solvent conditions were 0.1% formic acid in HPLC-grade water for the aqueous phase and 0.1% formic acid in acetonitrile for the organic phase. Drug spiked plasma calibrations were performed, with linear values ranging from 10 ng/ml to 10 μg/ml. The gradient ran from 10 to 100% acetonitrile for 5 min, maintained at 100% acetonitrile for 2 min, and from 100% to 10% for 3 min, resulting in a retention time of 3.2 min for dBET6.

### Quantification of dBET6 in NMC cells

NMC cells were plated in six-well plates at 80% confluency the day before treatment. The cells were then treated with either the free drug or the NP, at a final concentration of 1 μM dBET6. At the designated time points, the cells were trypsinized and harvested. Cells were protein-precipitated with 500 μl of cold acetonitrile before centrifugation at 4°C for 10 min at 12,700 rpm. The supernatant was dried and resuspended in 200 μl of acetonitrile for LC-MS analysis as described above.

### Quantification of dBET6 in tissue

NP biodistribution in NMC xenografts was quantified ex vivo using LC-MS. Tumor tissues were harvested at 4 hours postinjection. Tissues were weighed (100 to 150 mg), and each sample was placed into a homogenization tube with ceramic beads with 1.5 ml of acetonitrile. Tubes were run for 30 s at 6 m/s on a BeadMill24 (Thermo Fisher Scientific). Tubes were cooled on ice for 5 min before centrifugation at 12,700 rpm for 15 min at 4°C. The supernatant was dried and combined before completion. Following complete drying, 200 μl of acetonitrile was added and vortexed for 10 min before centrifugation under the same condition. The supernatant was carried on to LC-MS analysis as described above.

### Biodistribution studies of Fi-dBET6 and Fi-MS432 NPs

All animal studies were conducted according to protocols approved by the MSKCC Institutional Animal Care and Use Committee (IACUC). NMC (2 × 10^6^) cells or HCT116 (2.5 × 10^6^) cells were subcutaneously implanted in the hind limb 6- to 8-week-old Nu/Nu female athymic mice (Envigo). Cells were injected in a 1:1 solution of serum-free DMEM and Matrigel (BD Biosciences) in a total volume of 100 μl. The tumor models were used for biodistribution and tumor growth studies when the tumor reached 100 to 150 mm^3^. Imaging was done 24 hours after intraperitoneal treatment with Fi-dBET6 NPs (15 mg kg^−1^) or Fi-MS432 NPs (25 mg kg^−1^). Control mice were treated with untargeted dextran-wrapped NPs (Dex-dBET6 NPs or Dex-MS432 NPs). Mice were euthanized via CO_2_ inhalation and then dissected for ex vivo organ biodistribution. Images were taken using an iBright FL1500 imaging system. Fluorescence was captured after tumor excision with a 745-nm excitation filter and an 820-nm emission filter to observe NP emission. MFI was calculated for each region of interest using Fiji software.

### Pharmacokinetic studies

C57BL/6 female mice (6 to 8 weeks old) were treated intraperitoneally with either free dBET6 (40% Captisol in water) or Fi-dBET6 NPs (saline) at 15 mg/kg. Following single-dose administration, four mice from each group were bled at the following time points: 15 min, 30 min, 1 hour, 2 hours, 4 hours, and 8 hours. Mice were bled for a total of two times. A minimum of 250 μl of whole blood was collected into EDTA tubes after which plasma was separated and frozen for LC-MS analysis.

### In vivo xenograft studies

All mouse studies were conducted on IACUC-approved protocols (IACUC numbers 13-08-011 and 20-12-015) at MSKCC and followed all relevant ethical regulations. Tumor models were established as described above. After tumors reached 100 to 150 mm^3^, mice were randomized into treatment arms. dBET6 (15 mg kg^−1^ biweekly) or MS432 (25 mg kg^−1^ biweekly) was administered intraperitoneally. Intraperitoneal administration was chosen as the preferred method of injection due to the frequency of dosing and length of efficacy studies. Free drug and vehicle (DMSO) were formulated in 40% Captisol, and nanoPROTACs were resuspended in saline. Tumor volumes were calculated as (length × width^2^)/2.

### Statistics and reproducibility

Data reported are given as the mean ± SEM or mean with individual data points as indicated in the figure legends. Statistics were calculated using a paired or unpaired *t* test and two-way analysis of variance (ANOVA) with an appropriate post hoc test as stated in the figure legends. Survival plots of solid tumor models were analyzed using the Mantel-Cox log-rank test. Statistical significance was indicated accordingly: *P ≤ 0.05, **P ≤ 0.01, ***P ≤ 0.001, and ****P ≤ 0.0001. All statistical analyses were performed using GraphPad Prism version 9.1.0.
