# Supplementary material for: Tumor microenvironment–targeted PROTAC nanoparticle self-assembly broadly predicted by structural descriptors
Source: Sci Adv. 2025 Dec 5;11(49):eadu2292. doi: 10.1126/sciadv.adu2292 (PMC12680051; doi:10.1126/sciadv.adu2292)
Supplement: Supplementary file 1 — Figs. S1 to S18 [file sciadv.adu2292_sm.pdf]

Science Advances
AAAS

Supplementary Materials for

Tumor microenvironment–targeted PROTAC nanoparticle self-assembly broadly predicted by structural descriptors

Kristen C. Vogt *et al.*

Corresponding author: Daniel A. Heller, hellerd@mskcc.org

Sci. Adv. **11**, eadu2292 (2025)
DOI: 10.1126/sciadv.adu2292

This PDF file includes:

Figs. S1 to S18

Computational Library

A

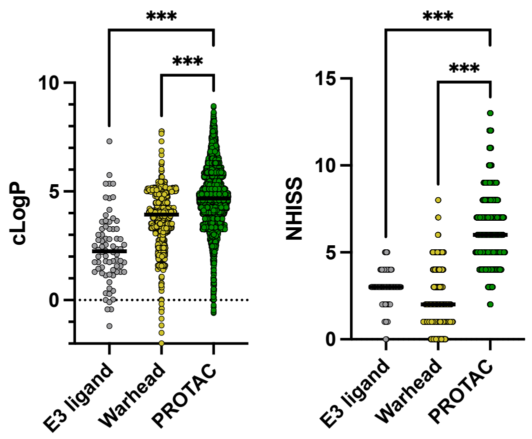

	cLogP	NHISS
E3 Ligand	3.7 ± 1.2	2.9 ± 1
Warhead	2.3 ± 1.6	2.1 ± 1.6
PROTAC	4.7 ± 1.4	6.3 ± 2

B

Validation Library

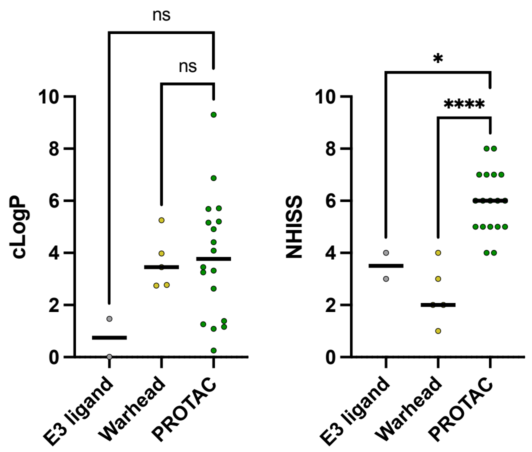

	cLogP	NHISS
E3 Ligand	0.74 ± 1.0	3.5 ± 0.7
Warhead	3.6 ± 1.0	2.4 ± 1.1
PROTAC	3.8 ± 2.3	5.9 ± 1.2

Fig. S1. NHISS and logP calculations for **(A)** a computational library using PROTAC-DB and **(B)** validation library of selected commercially available compounds. Scatter plots represent individual drugs in each library with a line at the median. Statistics were calculated using ordinary one-way ANOVA with Tukey's post hoc test. Values in both tables represent mean ± SD.

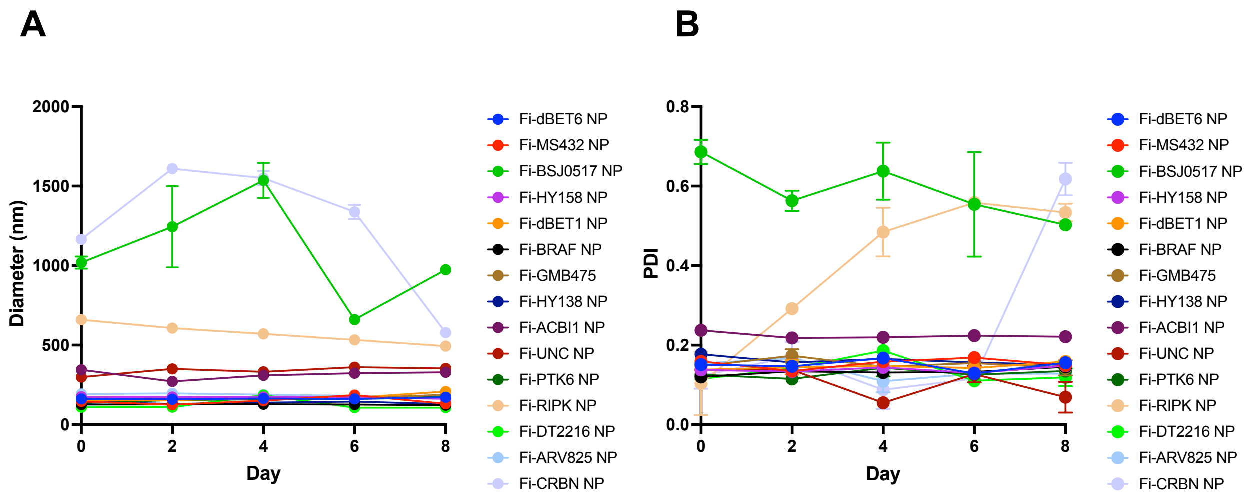

Figure S2. Determination of nanoparticle size cutoff. Stability of nanoPROTACs over time at 4°C, as measured by DLS **(A)** diameter and **(B)** PDI. Data are means of technical replicates ± SEM where n =3.

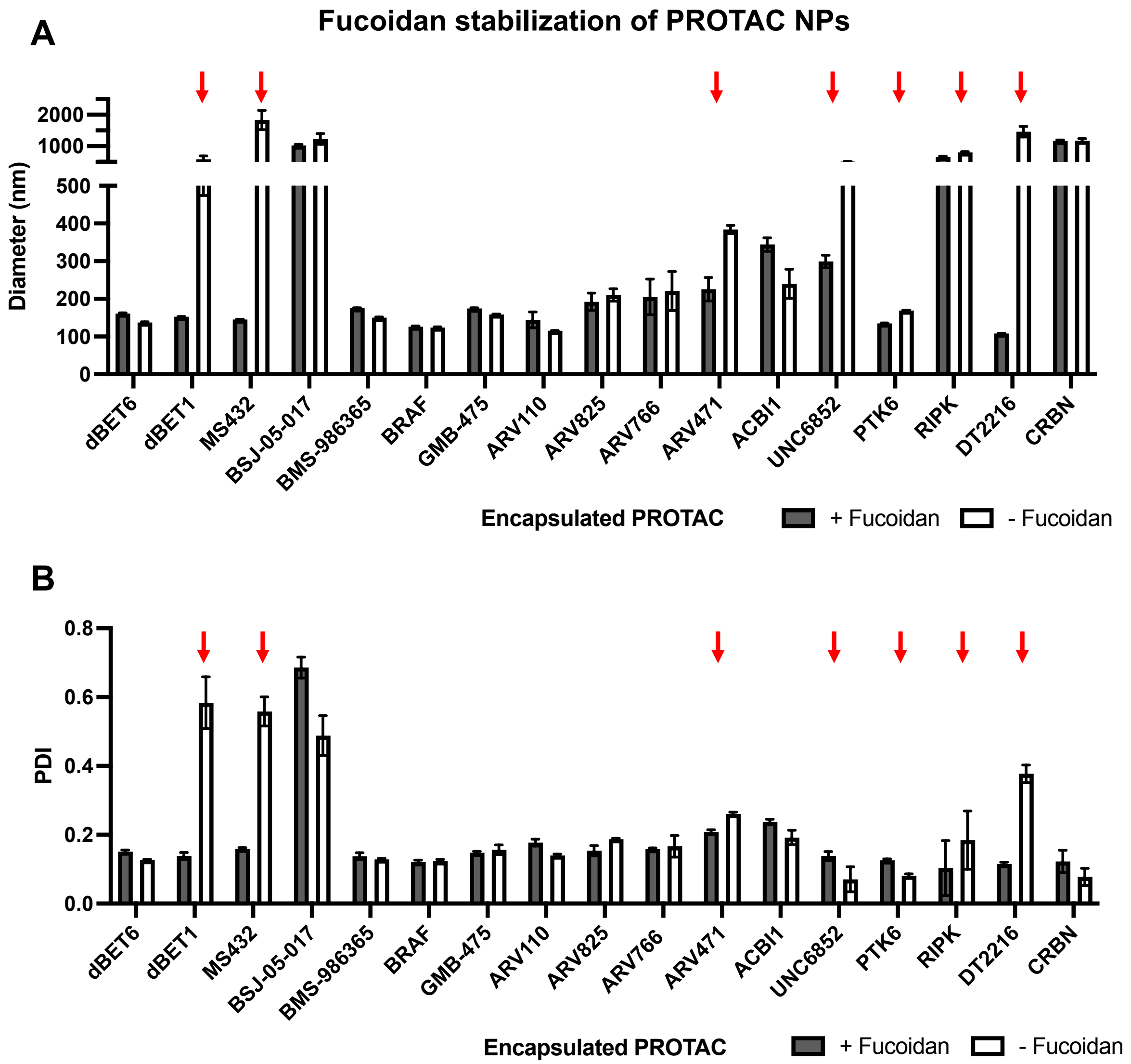

Fig. S3. Assessment of fucoidan in stabilizing nanoPROTACs. **(A)** Hydrodynamic diameter and **(B)** polydispersity index (PDI) of nanoPROTACs formulated with and without fucoidan, as measured by DLS. Data are means of technical replicates ± SD where n =3. Red arrows indicate drugs with improved stability upon fucoidan functionalization, as determined by decreased PDIs and hydrodynamic diameters.

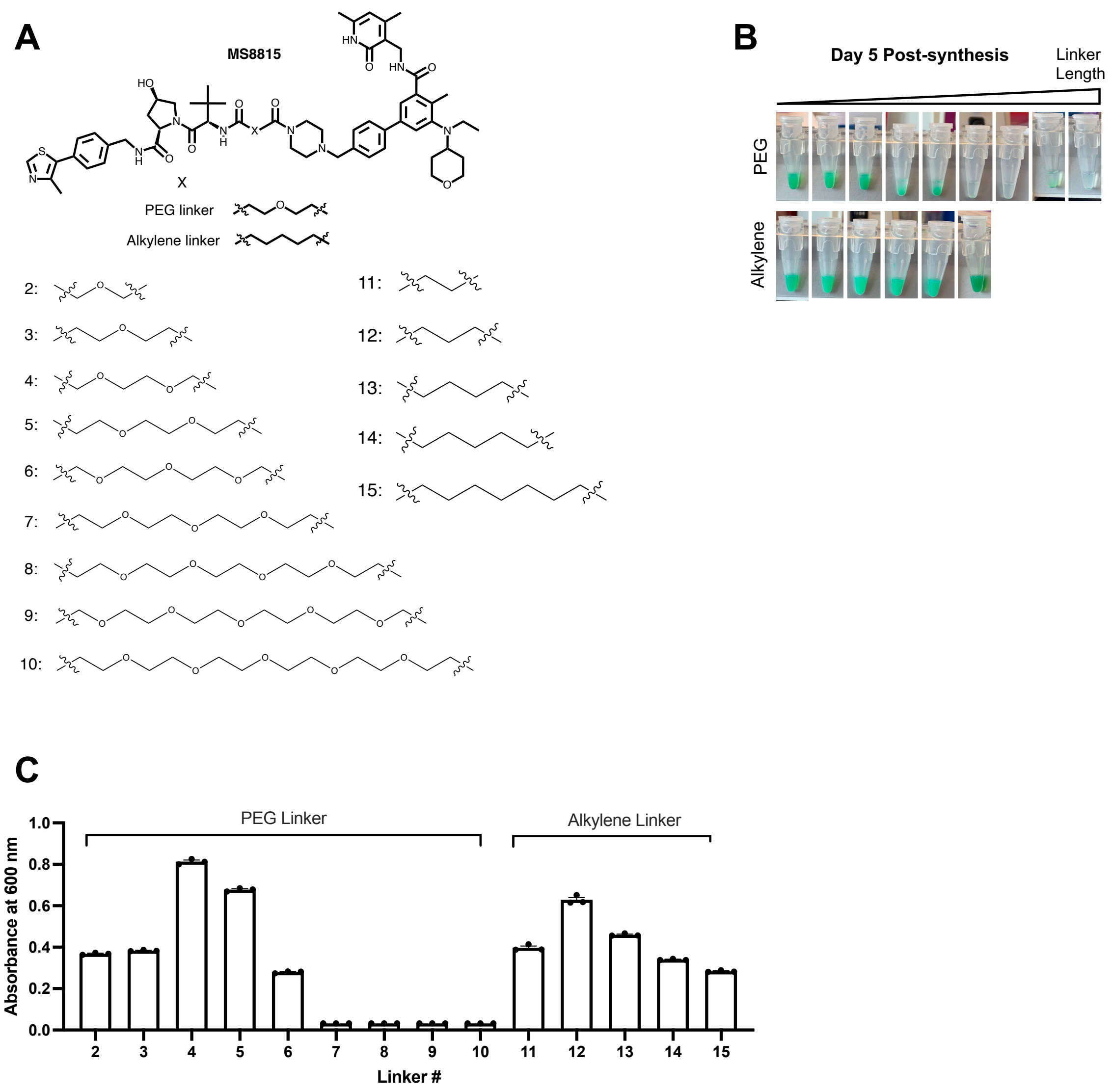

Fig. S4. Propensity for nanoformulation of EZH2 PROTACs via structure-nanoparticle assembly relationship (SNAR). (A) Structures of EZH2-targeted PROTACs with increasing linker length and varying composition. **(B)** Nanoparticle suspensions for each linker variant tested five days post-synthesis **(C)** Turbidity measurements of nanoparticles five days post-synthesis. NanoPROTACs with absorbance < 1 have precipitated and with absorbance > 1 are colloidally stable in solution. Data are means of technical replicates ± SD where n =3.

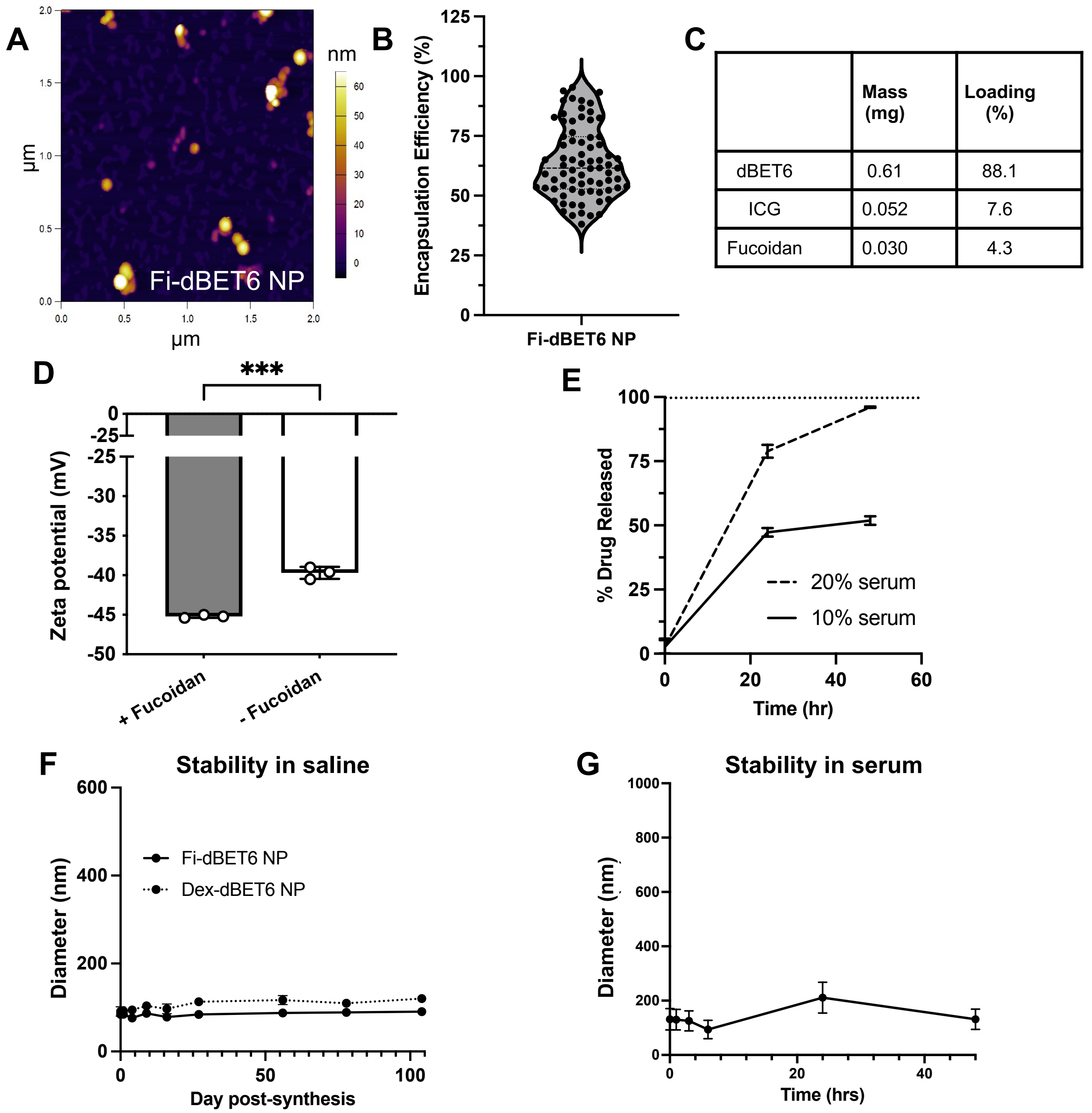

	Mass (mg)	Loading (%)
dBET6	0.61	88.1
ICG	0.052	7.6
Fucoidan	0.030	4.3

Fig. S5. Characterization of Fi-dBET6 NPs. (A) Atomic force micrograph of Fi-dBET6 NPs. **(B)** Summary of Fi-dBET6 NP encapsulation efficiency, as measured by HPLC (average = 64 ±15%, n = 75 biological replicates). **(C)** Complete composition of Fi-dBET6 NPs, as reported by percent drug loading. **(D)** Characterization of fucoidan functionalization, as measured by surface zeta potential. **(E)** Drug release profile of dBET6 from Fi-dBET6 NPs in 10% or 20% mouse serum at 37°C, as measured by HPLC. **(F)** DLS characterization of NanodBET6 particles at storage conditions (in saline at 4°C). **(G)** Fi-dBET6 NP stability in 25% mouse serum at 37°C, as measured by number mean DLS measurements. Data are means of technical replicates ± SD (C, D, E) or ± SEM (F) where n=3. Statistics were calculated using unpaired *t* test (D).

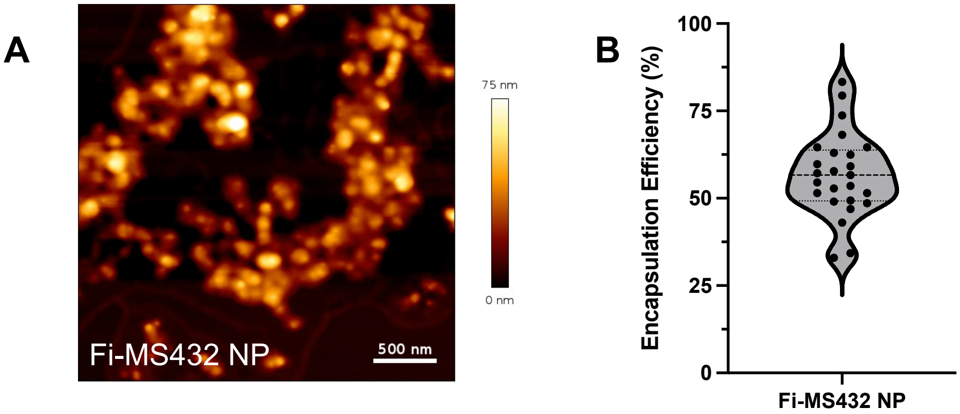

C

	Mass (mg)	Loading (%)
MS432	0.86	89.6
ICG	0.052	5.43
Fucoidan	0.047	4.94

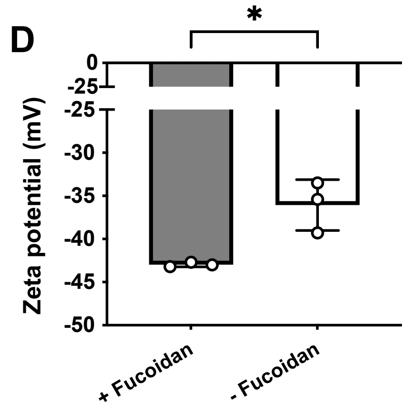

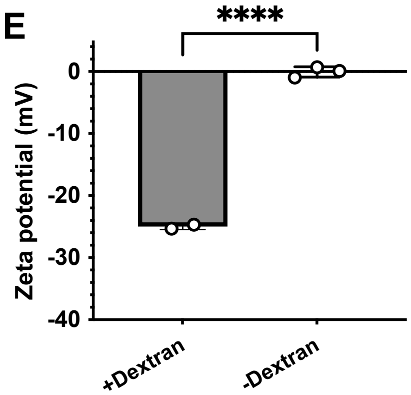

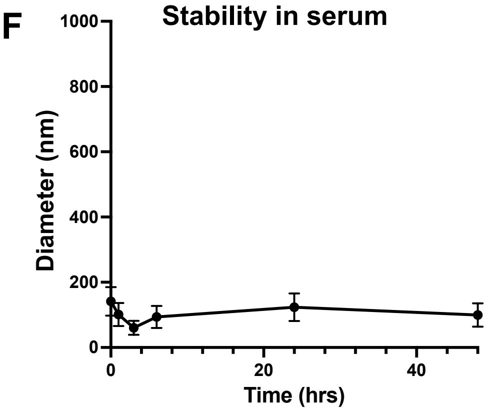

Fig. S6. Characterization of Fi-MS432 NPs. (A) Atomic force micrograph of Fi-MS432 NPs. **(B)** Summary of MS432 encapsulation efficiency (EE). Average EE = 56.7 ± 12.0% (n = 25 biological replicates). **(C)** Complete composition of Fi-MS432 NPs, as reported by percent drug loading. **(D)** Surface zeta potential of Fi-MS432 NPs with and without fucoidan wrapping. **(E)** Characterization of dextran functionalization, as measured by surface zeta potential. **(F)** DLS characterization of Fi-MS432 NPs at physiological conditions (in 25% mouse serum at 37°C). Data are means of technical replicates ± SD (B) or ± SEM (D) where n=3. Statistics were calculated using unpaired *t* test (D, E).

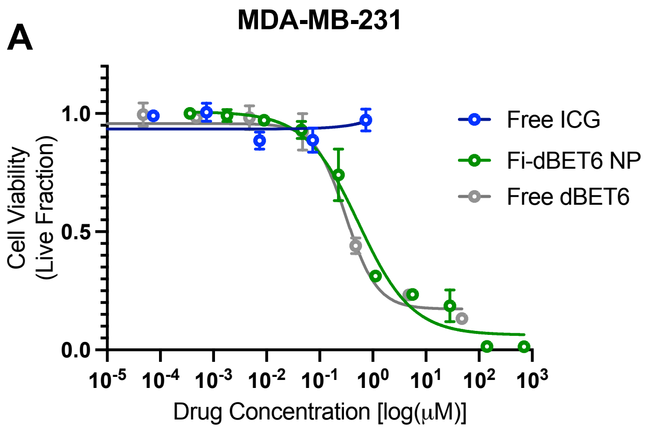

B

Formulation	IC_{50} (nM)
dBET6 FD	311
dBET6 ICG NP	542
ICG dye	N/A

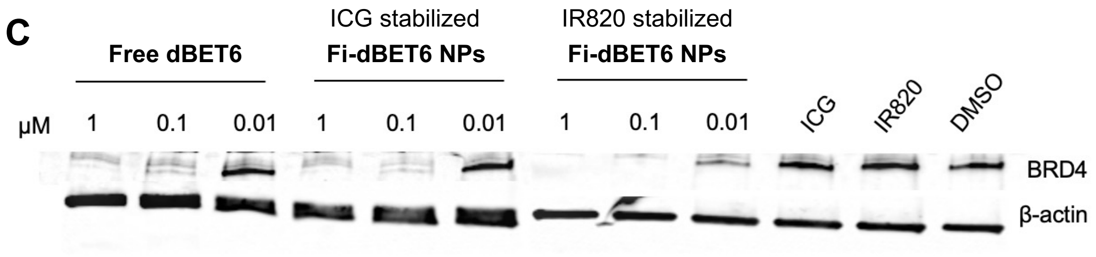

Fig. S7. Fi-dBET6 NPs maintain degrader functionality *in vitro*. Cell viability curves of MDA-MB-231 cells after treatment with **(A)** free dBET6 or Fi-dBET6 NPs, or dye only control, as measured by CellTiter-Glo. **(B)** Summary table of half maximal inhibitory concentration (IC_{50}) values in **(A)**. **(C)** Immunoblotting of BRD4 and b-actin in MDA-MB-231 cells after 24 h treatment. Data are means of technical replicates ± SD (A) where n=3.

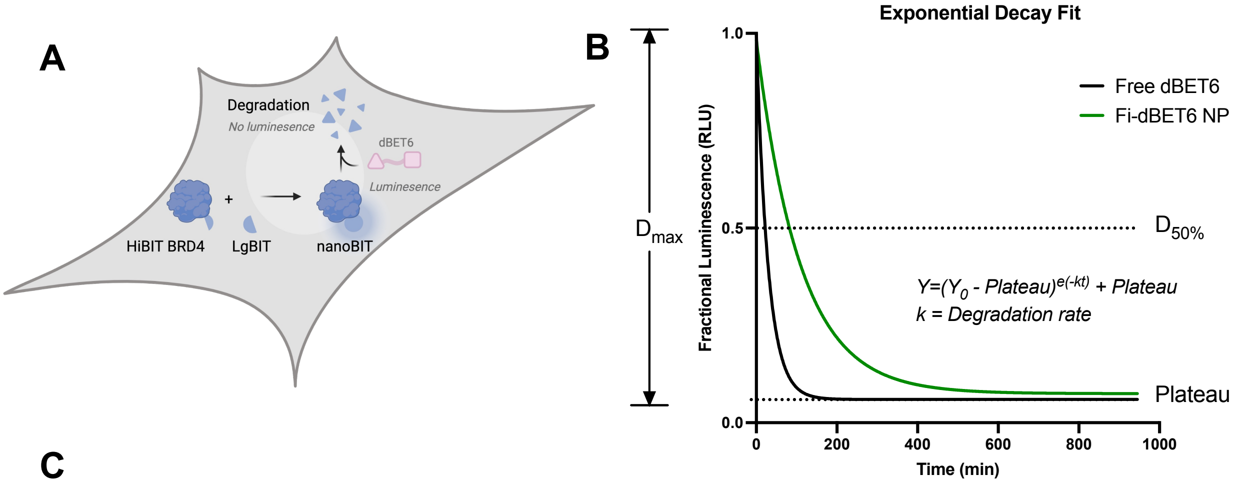

C

	Half life (min)	Plateau	Rate (min^{-1})	Tau (min)
Fi-dBET6 NP	75.2 ± 5.1 ****	0.06 ± 0.004 **	0.009 ± 0.001 ***	108 ± 4.4 ****
Free dBET6	20.2 ± 2.3	0.075 ± 0.003	0.034 ± 0.004	29.2 ± 3.3

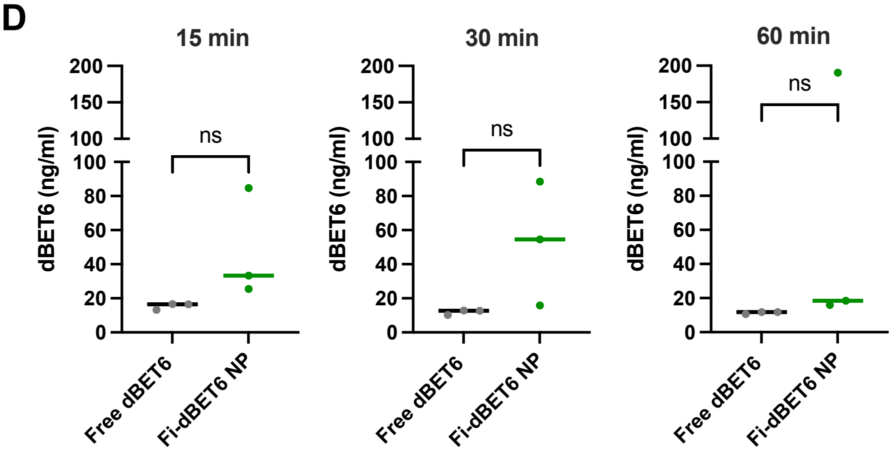

Fig. S8. Kinetics of intracellular target degradation and drug uptake (A) Schematic of NanoBIT platform in HEK293 background. HiBIT was inserted via CRISPR/Cas9 to the N-terminus of BRD4 in HEK293 cell line stably expressing LgBIT, which complements with HiBIT tag to form luminescent NanoBIT luciferase. Exponential decay fit **(B)** and kinetic parameters **(C)** calculated from Figure 2c. **(D)** Intracellular dBET6 concentrations in NMC cells post-washout, as measured by LC/MS. Tau is defined as the time constant, or the reciprocal of the rate constant (k). Data are individual biological replicates (D) or means of technical replicates ± SD (C) where n=3. Statistics were calculated using unpaired *t* test (C).

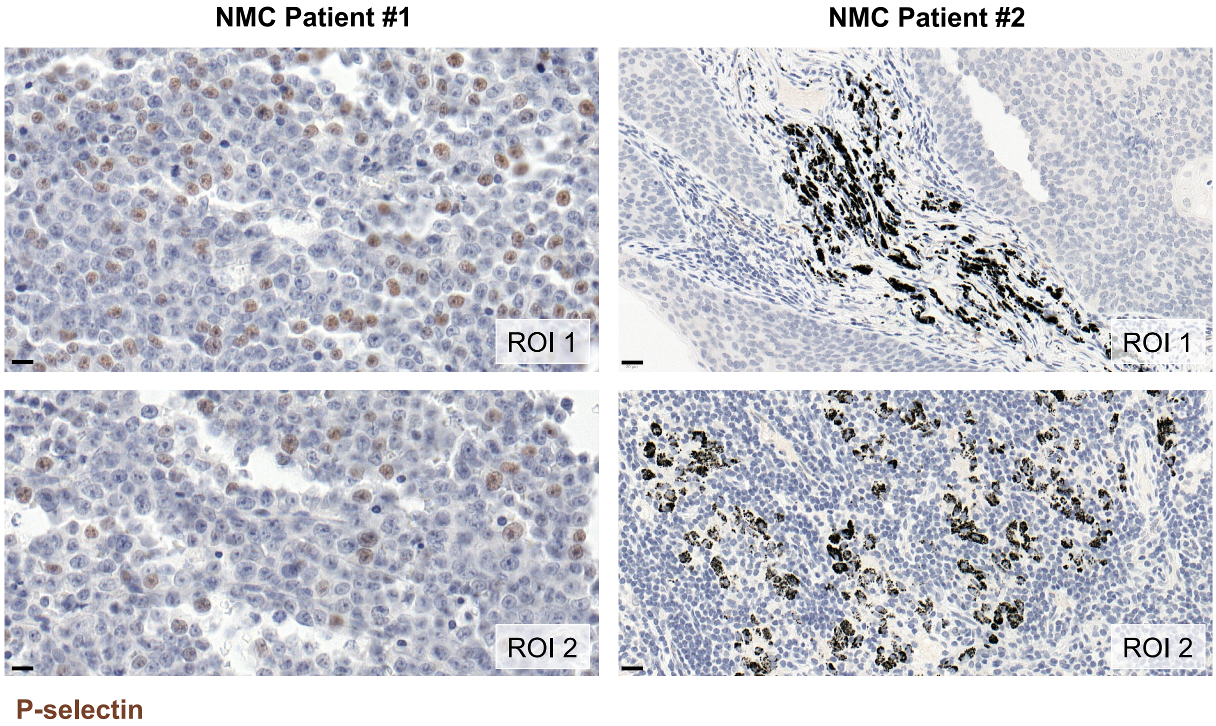

Fig. S9. Primary human NMC tumors express P-selectin. Immunohistochemistry of P-selectin in formalin-fixed, paraffin-embedded primary patient tumor slides.

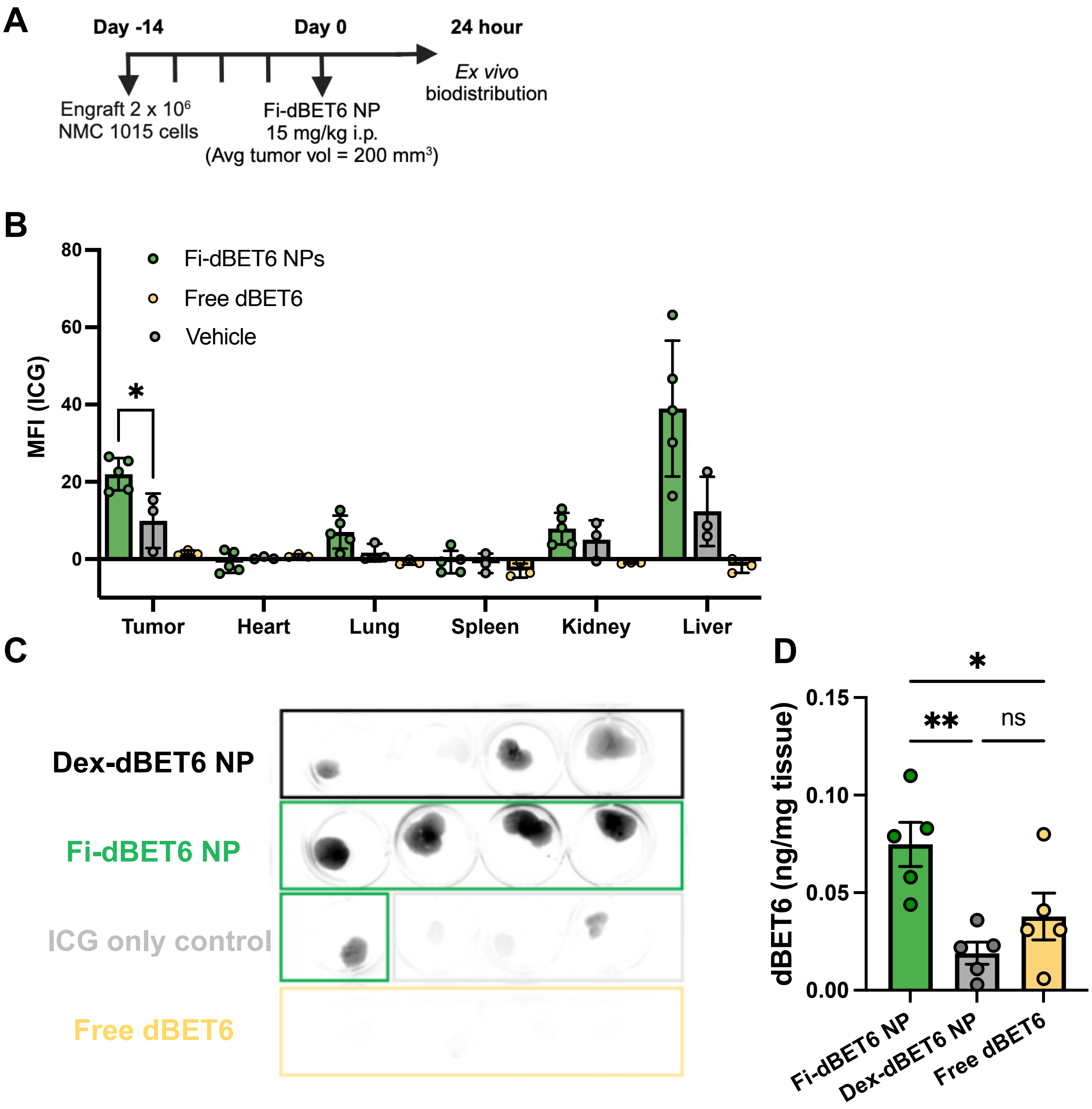

Fig. S10. Fi-dBET6 NPs accumulate in subcutaneous NMC tumors. **(A)** Schema of biodistribution setup **(B)** Biodistribution of Fi-dBET6 NPs or dye only control in tumor and normal tissue 24 h post-injection i.p. **(C)** Raw images of tumor tissue quantified in Figure 4B demonstrating accumulation of P-selectin-targeted versus dextran-untargeted NPs in tumor tissue 24 h post-injection i.p. **(D)** Concentration of dBET6 in tumor tissue 4 hours post-treatment (i.p.), as measured by LC-MS. Data are shown as individual biological replicates with means ± SEM and statistics were calculated using unpaired *t* test of Fi-dBET6 versus ICG only (B), or one-way ANOVA with Fisher's LSD test (D).

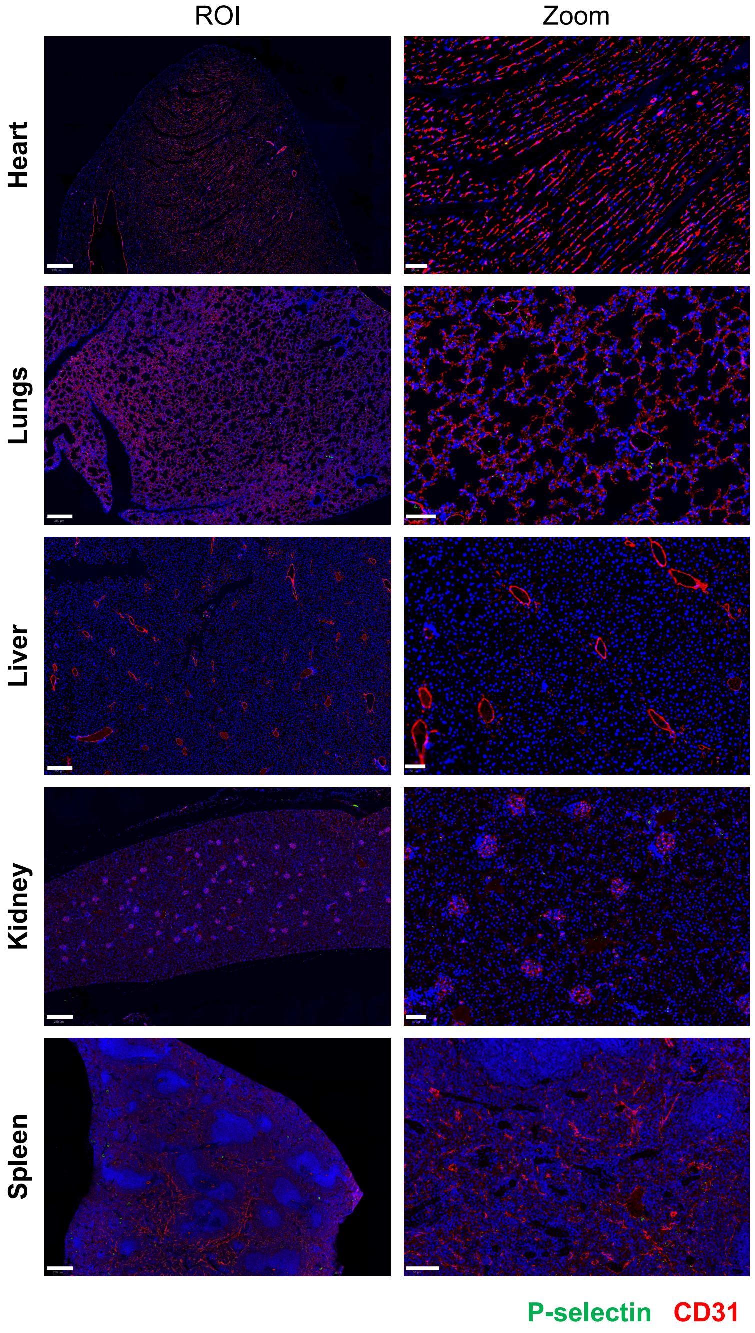

Fig. S11. P-selectin expression in normal murine tissue. Immunofluorescence staining of CD31 (red) and P-selectin (green) in heart, lung, liver, kidney and spleen of healthy female nude mice. One region of interest (ROI) is shown per tissue (scale bar 250 µm) with a zoomed in view to the right (scale bar 50 µm).

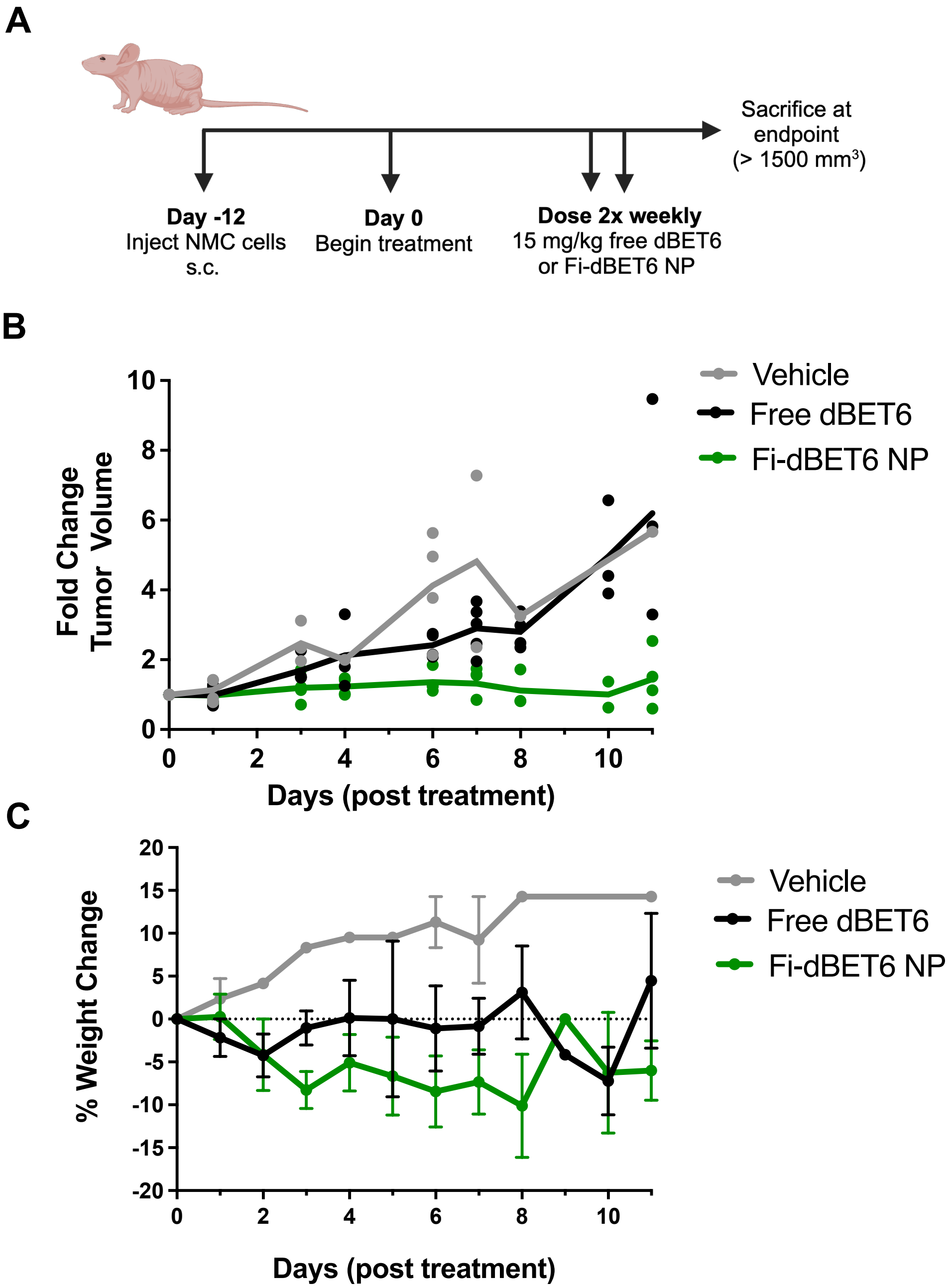

Fig. S12. Fi-BET6 NPs are efficacious in a pre-clinical murine model of NUT Midline Carcinoma (Replicate study). (A) Schematic of anti-tumor efficacy study. Nude mice engrafted subcutaneously with NMC cells were treated twice weekly with vehicle, free dBET6, or Fi-dBET6 NPs. Tumor burden was assessed by digital calipers until clinical endpoint (tumor burden > 1500 mm^3 or weight loss > 20%). **(B)** Fold change tumor volume curves of NMC xenografts treated biweekly with 15 mg/kg i.p. treatments of free dBET6, nanodBET6, vehicle or untreated. **(C)** Percent weight change normalized to day 0. n = 5 mice per treatment group; n = 4 mice vehicle. Data are individual biological replicates (B) or means ± SEM (C).

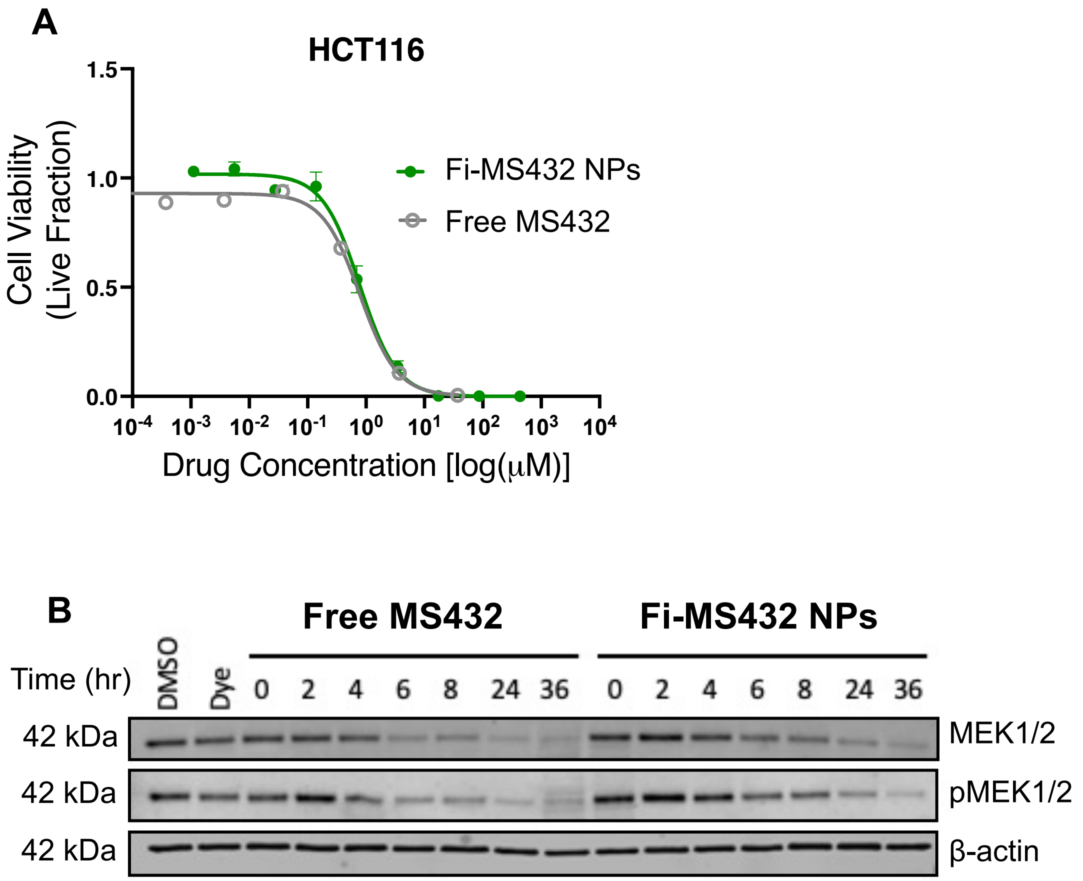

Fig. S13. Fi-MS432 NPs maintain degrader functionality in vitro. (A) Cell viability curves of HCT116 colorectal cancer cells after treatment with free or Fi-MS3432 NPs, as measured by CellTiter-Glo. **(B)** Immunoblotting of MEK1/2, pMEK1/2 and β-actin in HCT116 cells after varying incubation times with free MS432 of Fi-MS432 NPs. Data are means of technical replicates ± SD (A) where n=3.

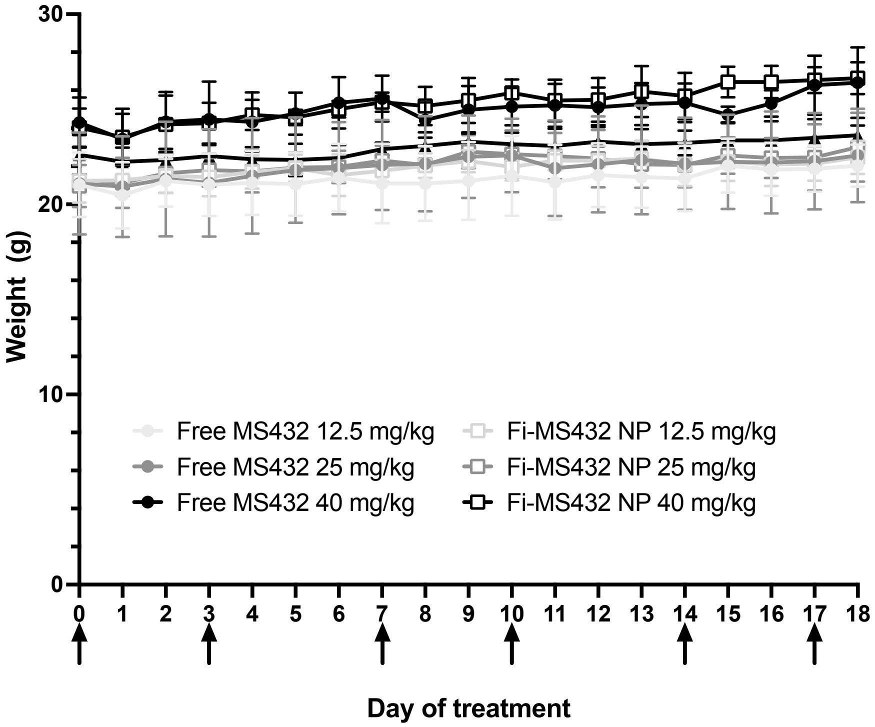

Fig. S14: Maximum tolerated dose escalation of MS432. Healthy nude mice were treated twice weekly i.p. with 12.5, 25 or 40 mg/kg free MS432 or Fi-MS432 NPs. Arrows denote days when mice were dosed. Data are means ± SD (n = 3 biological replicates per group).

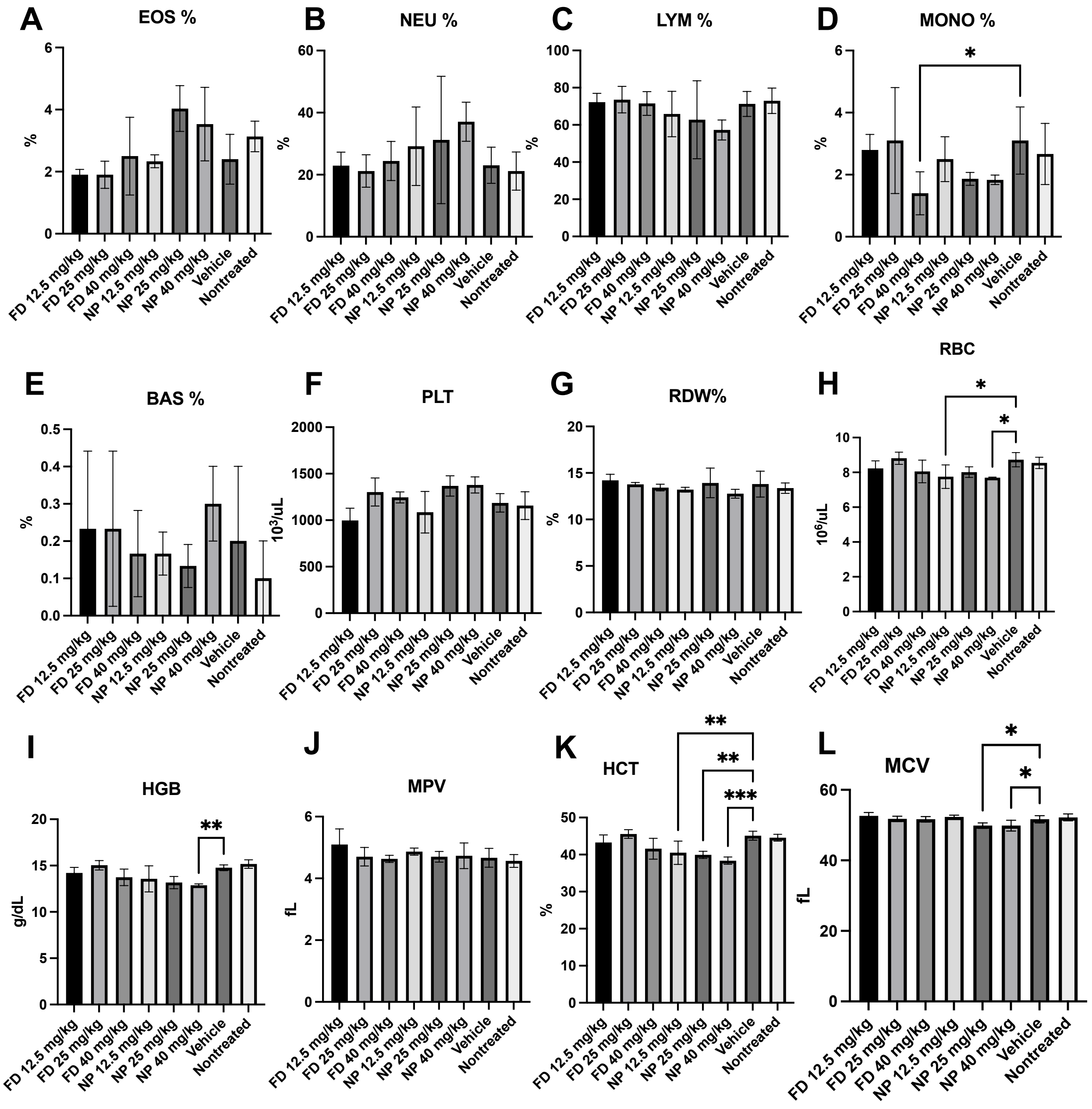

Fig. S15. Complete blood count toxicity assessment associated with MS432 MTD (Fig. S14). Blood was harvested by cheek bleed 24 hours after the final dose of MS432 (Day 18) and used for complete blood count to assess chronic treatment toxicity. **(A)** Eosinophil, **(B)** neutrophil, **(C)** lymphocyte, **(D)** monocyte, **(E)** basophil, **(F)** platelet, **(G)** red cell distribution width, **(H)** red blood cell, **(I)** hemoglobin, **(J)** mean platelet volume, **(K)** hematocrit percentage, **(L)** mean corpuscular volume (fL). Data are means ± SD (n = 3 biological replicates per group). FD = Free MS432 drug and NP = nanoparticle formulated MS432. Statistics were calculated using ordinary one-way ANOVA with Fisher's LSD test.

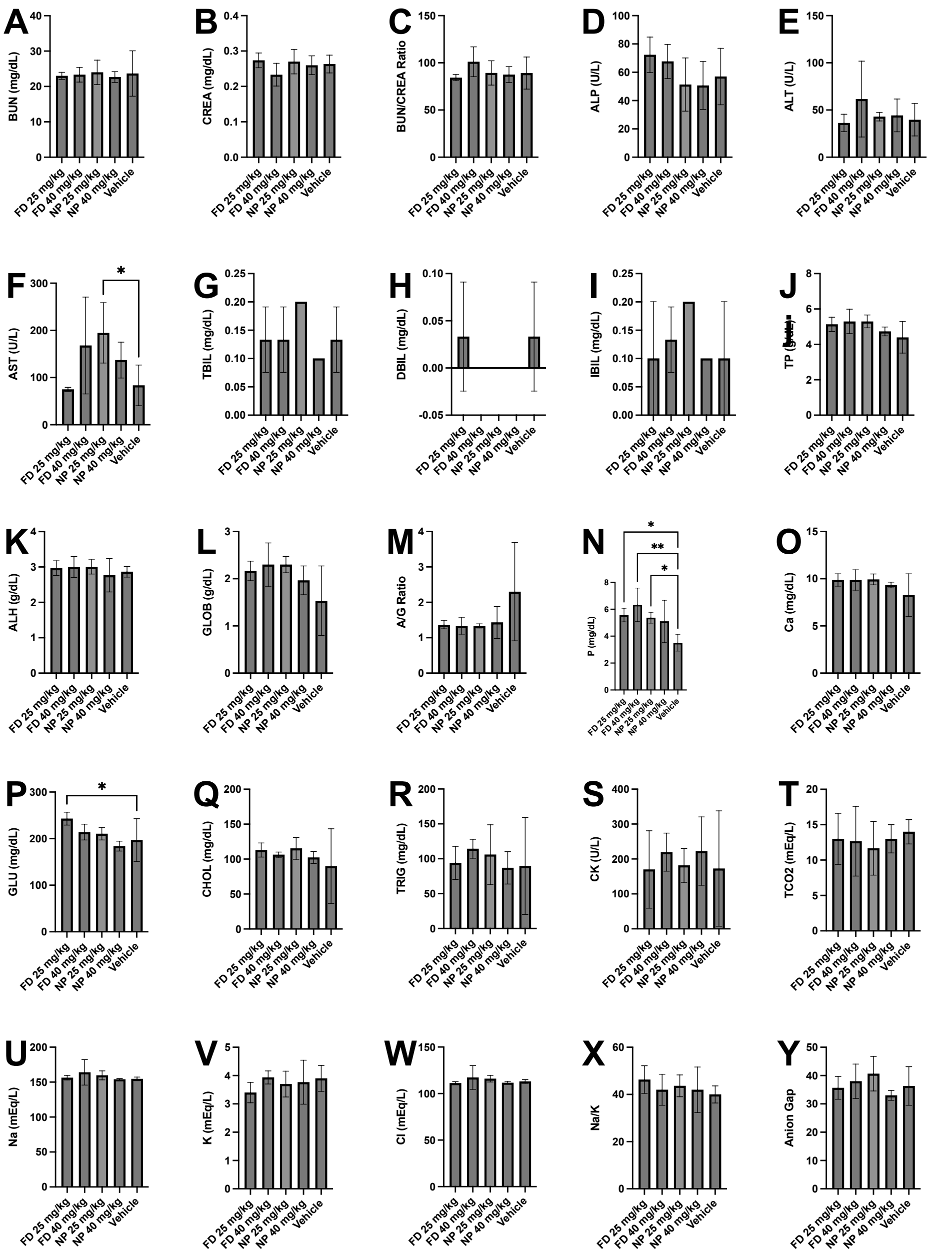

Fig. S16. Serum clinical chemistry panel toxicity following MS432 MTD study (Fig. S14). Blood was harvested by cheek bleed 24 hours after the final dose of MS432 (Day 18) and serum was used for the clinical chemistry panel to assess chronic treatment toxicity. **(A)** Blood urea nitrogen, **(B)** Creatine, **(C)** Ratio of blood urea nitrogen to creatine, **(D)** Alkaline phosphatase, **(E)** Alanine transaminase, **(F)** Aspartate aminotransferase, **(G)** Total bilirubin, **(H)** Direct bilirubin, **(I)** Indirect bilirubin, **(J)** Total protein, **(K)** ALH, **(L)** Globulin, **(M)** Albumin/globulin ratio, **(N)** Phosphate, **(O)** Calcium, **(P)** Glucose, **(Q)** Cholesterol, **(R)** Triglycerides. **(S)** Creatine kinase, **(T)** Carbon dioxide, **(U)** Sodium. **(V)** Potassium. **(W)** Chloride. **(X)** Sodium / potassium ratio. **(Y)** Anion gap. Data are means ± SD (n = 3 biological replicates per group). FD = Free MS432 drug and NP = nanoparticle formulated MS432. Statistics were calculated using ordinary one-way ANOVA with Fisher's LSD test.

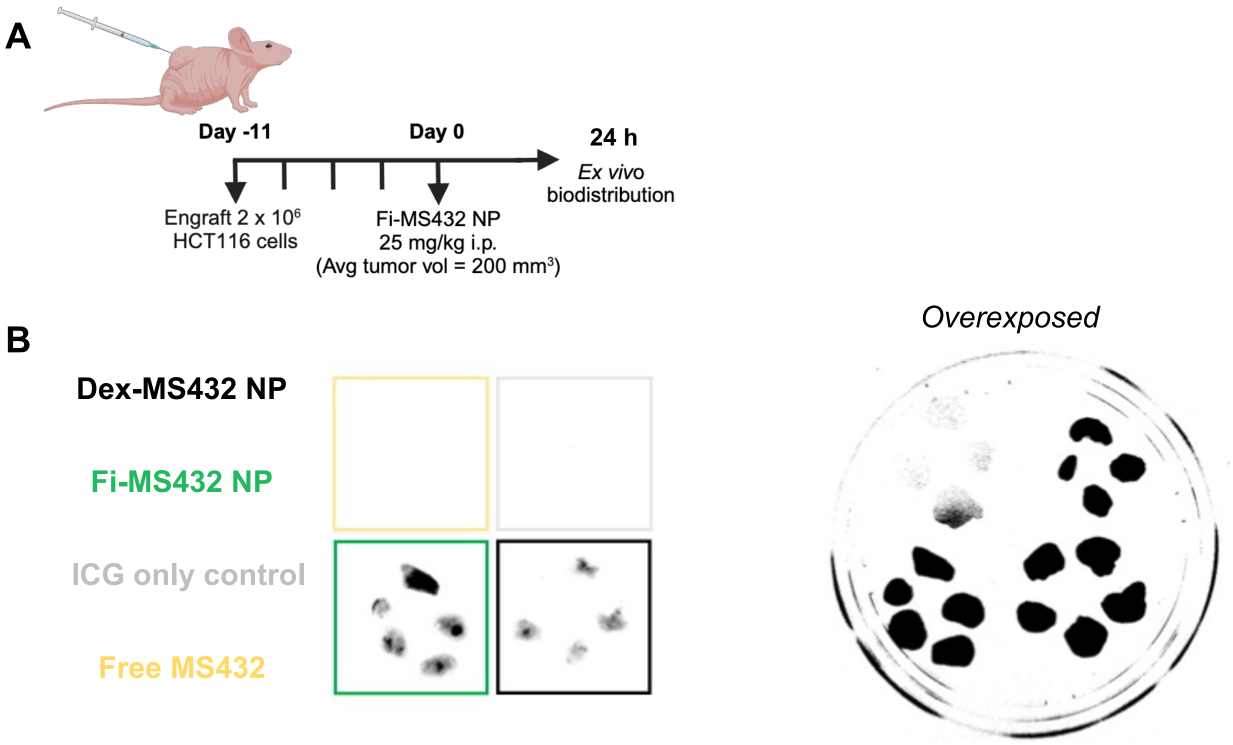

Fig. S17. Fi-MS432 NPs preferentially accumulate in HCT116 tumors over untargeted dextran NPs, as quantified in Fig. 5b. **(A)** Schema of biodistribution setup. **(B)** Accumulation of P-selectin-targeted versus dextran-untargeted NPs in tumor tissue 24 h post-injection i.p. Right image is overexposed to show tissue boundaries.

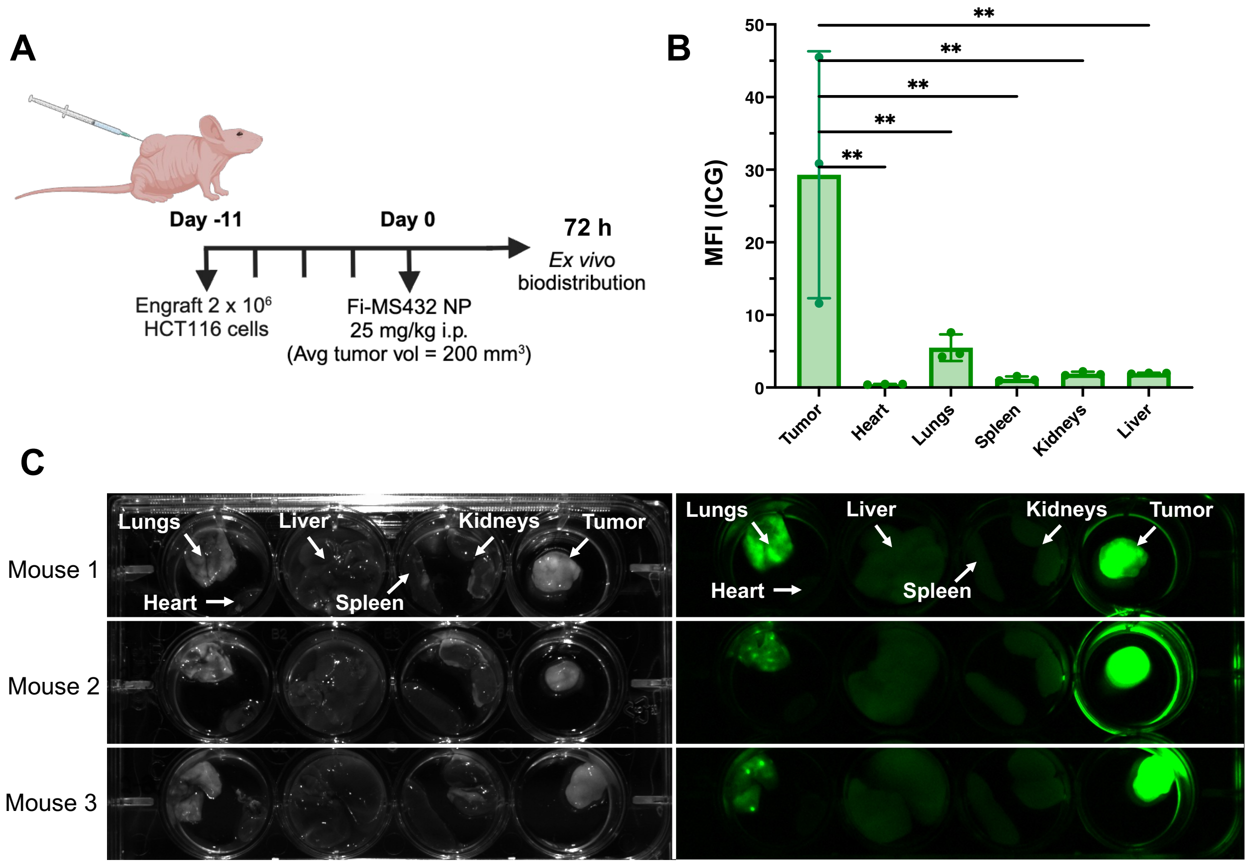

Fig. S18. Fi-MS432 NPs preferentially accumulate in HCT116 tumors over normal tissue. **(A)** Schema of biodistribution setup to assess retention of nanoparticles in healthy tissue. **(B)** Biodistribution of Fi-dBET6 NPs in tumor and normal tissue 72 h post-injection i.p., as measured by ICG mean fluorescence intensity (MFI) **(C)** Raw brightfield (left) and fluorescence (right) images of tumor and normal tissues, as quantified in b. Data are shown as individual biological replicates with means ± SD (B). Statistics were calculated using an ordinary one-way ANOVA with Dunnett's post hoc test.
